# Metallothionein 1B attenuates inflammation and hepatic steatosis in MASH by inhibiting the AKT/PI3K pathway

**DOI:** 10.1016/j.jlr.2024.100701

**Published:** 2024-11-16

**Authors:** Canghai Guan, Xinlei Zou, Wujiang Shi, Jianjun Gao, Chengru Yang, Yifei Ge, Zhaoqiang Xu, Shaowu Bi, Xiangyu Zhong

**Affiliations:** 1 General Surgery Department, The 2nd Affiliated Hospital of Harbin Medical University, Harbin, Heilongjiang Province, China; 2 The Key Laboratory of Myocardial Ischemia, Harbin Medical University, Ministry of Education, Harbin, Heilongjiang, China

**Keywords:** metabolic dysfunction, associated steatohepatitis, MT1B, AKT/PI3K, MTF1, zinc

## Abstract

Metabolic dysfunction–associated steatohepatitis (MASH) is a severe form of metabolic dysfunction–associated fatty liver disease metabolic dysfunction-associated steatohepatitis , characterized by hepatic steatosis, inflammation, and fibrosis. This study investigates the role and potential mechanisms of metallothionein 1B (MT1B) in MASH through bioinformatics analysis and experimental validation. quantitative reverse transcription PCR and Western blot analyses confirm that MT1B expression is significantly downregulated in liver tissues of MASH patients, in high-fat diet–induced mouse models, and in hepatocytes induced by FFAs. Further functional experiments show that upregulation of MT1B reduces intracellular triglycerides and total cholesterol levels, lipid droplet formation, and proinflammatory factors. In vivo experiments demonstrate that specific downregulation of hepatic MT1B expression via AAV8-shMT1B injection significantly increases triglyceride and total cholesterol levels, exacerbates lipid accumulation, and markedly elevates liver fibrosis and inflammatory factor expression. RNA-seq and bioinformatics analyses show that the AKT/PI3K pathway is significantly suppressed in MT1B-overexpressing cells. Further experiments indicate that AKT inhibition can reverse the lipid metabolism disorders and inflammatory responses caused by MT1B downregulation. Additionally, Zinc can promote the nuclear translocation of MTF1, leading to its binding to the MT1B promoter, thereby upregulating MT1B expression and ultimately mitigating MASH progression. These findings suggest that zinc-regulated MT1B plays a critical role in lipid metabolism and inflammatory responses by regulating the AKT/PI3K signaling pathway, influencing MASH progression.

## Graphical Abstract

**Figure:**
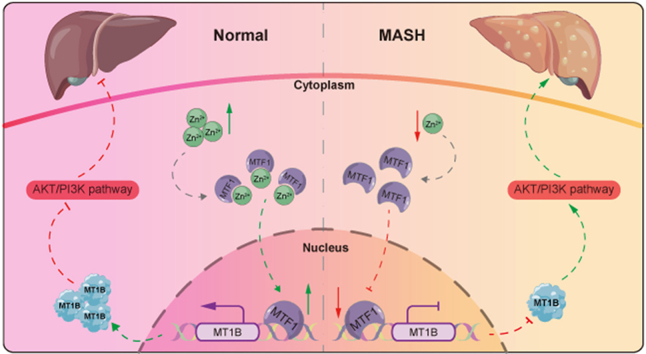

Metabolic dysfunction–associated fatty liver disease (MAFLD) is one of the most prevalent chronic liver disorders globally, with its prevalence rapidly increasing over the past few decades (1, 2, 3, 4). This rising trend is primarily driven by the global epidemics of obesity, metabolic syndrome, and type 2 diabetes (5, 6, 7, 8, 9, 10). MAFLD encompasses a spectrum ranging from simple steatosis to the more severe form known as metabolic dysfunction–associated steatohepatitis (MASH), which can further progress to liver fibrosis, cirrhosis, and even hepatocellular carcinoma (11, 12, 13, 14). The pathological characteristics of MASH include hepatic steatosis, inflammation, and fibrosis (15, 16, 17, 18). Its pathogenesis involves complex metabolic disorders, lipid accumulation, oxidative stress, and chronic inflammation (19, 20). However, effective therapeutic options for MASH remain scarce, emphasizing the urgent need to elucidate its underlying molecular mechanisms to identify potential therapeutic targets.

Metallothioneins (MTs) are a class of low-molecular-weight, metal-binding proteins that play important roles in metal ion metabolism, oxidative stress protection, and cell proliferation (21). The MT family is classified into four main isoforms: MT1, MT2, MT3, and MT4 (22). MTs are characterized by their high cysteine content, which enables them to bind metal ions (such as zinc, copper, and cadmium), thereby contributing to metal ion homeostasis, metal detoxification, and protection against oxidative stress–induced cellular damage (23). MT1 and MT2 are primarily expressed in the liver and kidneys, while MT3 and MT4 are mainly found in the nervous system and epithelial cells (24, 25, 26, 27). Metallothionein 1B (*MT1B*) is a member of the MT1 subtype, and recent studies have shown that MT1B plays important roles in various physiological and pathological processes (28). For instance, MT1B expression is upregulated in response to exposure to heavy metals such as cadmium, lead, and mercury, highlighting its critical role in metal detoxification (29). Additionally, MT1B can mitigate oxidative damage by scavenging free radicals, thereby functioning as a key antioxidant (30). Nonetheless, the specific functions and regulatory mechanisms of MT1B in MASH remain poorly understood. Therefore, this study aims to investigate MT1B expression alterations in MASH and its impact on lipid metabolism and inflammatory responses, further elucidating its potential molecular mechanisms.

This study found that MT1B expression is significantly lower in MASH tissues and cell samples than normal tissues and cells. In vitro and in vivo experiments confirmed that MT1B downregulation leads to increased intracellular triglyceride (TG) and total cholesterol (TC) levels, significantly increased lipid droplet formation, and markedly upregulated expression of proinflammatory factors. MT1B exerts its protective role by inhibiting the activation of the AKT/PI3K signaling pathway. Additionally, zinc promotes the nuclear translocation of MTF1, which, as a transcription factor promoter of MT1B, can upregulate MT1B expression, thereby mitigating the progression of MASH. These findings suggest that MT1B plays critical roles in lipid metabolism and inflammatory responses, influencing the progression of MASH through the regulation of the AKT/PI3K signaling pathway. At the same time, Zn regulates MASH progression by affecting the binding of MTF1 to MT1B, providing new perspectives and directions for MASH treatment.

## Materials and methods

### Clinical samples

Clinical samples in this study were obtained from MASH patients (n = 10) and healthy controls (n = 10) who underwent liver biopsy. All patients signed informed consent forms, and sample collection was approved by the Ethics Committee of The Second Affiliated Hospital of Harbin Medical University. Inclusion criteria were 1. age 18–65 years; 2. diagnosed with MASH through pathological diagnosis and clinical laboratory tests; and 3. voluntary signing of informed consent. Exclusion criteria included 1. severe diseases of major organs such as the heart, liver, or kidneys; 2. malignant tumors; 3. pregnancy or lactation; and 4. history of mental illness. Some voluntary patients agreed to undergo liver biopsy, and liver tissue samples were obtained according to standard procedures. Part of the samples was used for pathological diagnosis, and the remaining parts were rapidly frozen with liquid nitrogen and stored at −80°C until use. The samples were used for qRT-PCR and Western blot analysis to determine MT1B expression. All procedures performed in studies involving human participants were in accordance with the ethical standards of the institutional and national research committee and with the Declaration of Helsinki and its later amendments or comparable ethical standards. All patients provided informed consent, and the Ethics Committee of The Second Affiliated Hospital of Harbin Medical University approved this study (YJSKY2023-149). Clinicopathological characteristics of participants were listed in Supplementary Table 1.

### Cell culture and transfection

Human liver cell HepG2 and mouse liver cell AML12 were obtained from the Cell Bank of the Chinese Academy of Sciences (Shanghai, China). HepG2 cells were cultured in high-glucose DMEM (Gibco, New York) containing 10% FBS (Gibco). AML12 cells were cultured in DMEM/F-12 medium (Gibco) containing 10% FBS and 1× insulin-transferrin-selenium (Sigma-Aldrich, St. Louis, MO). Cells were maintained at 37°C in a 5% CO_2_ atmosphere. For FFA treatment, oleic acid (Sigma-Aldrich, Cat. No. O1008) and palmitic acid (Sigma-Aldrich, Cat. No. P0500) were prepared in a 2:1 ratio with final concentrations of 400 μM and 200 μM, respectively. FFA was first dissolved in ethanol and then conjugated to 5% bovine serum albumin (Thermo Fisher Scientific, Cat. No. BP1600) by adding to prewarmed BSA solution at 37°C with continuous stirring until fully dissolved, forming stable FFA-BSA complexes. These complexes were then added to the cell culture medium for subsequent treatments. As a control, BSA was added at a final concentration of 5% in the medium. Transfection experiments were performed using Lipofectamine 2000 (Invitrogen, Carlsbad, CA). For MT1B knockdown experiments, MT1B-specific siRNA (GenePharma, Shanghai, China) and nontargeting control siRNA were used. For MT1B overexpression experiments, a human MT1B gene was cloned into the pcDNA3.1 (+) vector (Invitrogen, Cat. No. V79020) and an empty vector control were used. Cells were seeded in 6-well plates, and transfection was carried out when cell confluency reached 70%–80%. siRNA or plasmid vectors were mixed with Lipofectamine 2000 and incubated for 20 min to form complexes, which were then added to the cell culture medium and gently mixed. Cells were cultured for an additional 48 h posttransfection. After the initiation of FFA treatment, si-MT1B + DMSO, si-MT1B + MK-2206 (MedChemExpress, New Jersey, Cat. No. HY-10358) and si-MT1B + ZnSO_4_ (MedChemExpress, Cat. No. HY-N3025) were added 24 h later. The final concentration of MK-2206 and ZnSO_4_ were 5 μM and 10 μM, respectively, and the treatment continued for 24 h. Treated cells were used for qRT-PCR, Western blot, Oil red O staining, Nile red staining, and measurements of TG and TC levels.

### qRT-PCR

Total RNA was extracted from cells and tissue samples using TRIzol reagent (Invitrogen) according to the manufacturer's instructions. The purity and concentration of the extracted RNA were determined by agarose gel electrophoresis and a NanoDrop 2000 (Thermo Fisher Scientific, Waltham, MA). cDNA synthesis was performed using a reverse transcription kit (Roche, Penzberg, Germany) according to the manufacturer's instructions. Real-time quantitative PCR was conducted using a C1000 Thermal Cycler (Bio-Rad, Hercules), and the reaction system was prepared according to the SYBR Green PCR Master Mix (Roche) instructions. Primer sequences are listed in Supplementary Table 2. The qRT-PCR reaction conditions were: initial denaturation at 95°C for 10 min, followed by 40 cycles of 95°C for 15 s, 60°C for 30 s, and 72°C for 30 s. Each sample was tested in triplicate, GAPDH was used as the reference gene for normalization and relative expression levels were calculated using the 2^-ΔΔCT^ method.

### Western blot

Cells and tissue samples were lysed in RIPA lysis buffer (Beyotime, Shanghai, China) with protease inhibitors (Beyotime), and total protein concentrations were determined using a BCA Protein Assay Kit (Beyotime). Equal amounts of protein samples were separated by SDS-PAGE and transferred onto PVDF membranes (Millipore, Billerica, MA). Membranes were blocked with 5% nonfat milk (Beyotime) at room temperature for 1 h and then incubated overnight with primary antibodies. Antibodies used for Western bolt are listed in Supplementary Table 3. Subsequently, membranes were incubated with HRP-conjugated secondary antibodies at room temperature for 1 h. Bands were visualized using ECL detection reagents (Thermo Fisher Scientific), photographed using image analysis system (Bio-Rad), and band intensities were analyzed with ImageJ software (NIH, Bethesda, MD). GAPDH was used as an internal control.

### TG and TC detection

The levels of TG and TC in cell and liver tissue samples were measured using commercial assay kits (Nanjing Jiancheng Bioengineering Institute, Nanjing, China). According to the kit instructions, cell or tissue samples were homogenized and lysed in PBS (Gibco), followed by centrifugation to collect the supernatant. TG and TC contents were determined using TG and TC assay kits, respectively. The specific steps included enzymatic reactions, color development, and reading absorbance at 500 nm on a microplate reader (Thermo Fisher Scientific). TG and TC contents in the samples were calculated using standard curves.

### Oil red O staining

Oil red O staining was used to detect intracellular lipid droplet accumulation. Cells were cultured and treated in 6-well plates and then washed twice with PBS and fixed with 4% paraformaldehyde (Sigma-Aldrich) for 30 min. After fixation, cells were washed with 60% isopropanol (Sigma-Aldrich) and stained with Oil red O solution (Solarbio, Beijing, China) for 10 min. Excess stain was removed with 60% isopropanol, and cells were rinsed three times with PBS. Cells were observed and photographed under a microscope (Olympus, Tokyo, Japan).

### Nile red staining

Nile red staining was used to detect intracellular neutral lipid accumulation. Treated cells were washed twice with PBS, and then fixed with 4% paraformaldehyde for 30 min. After fixation, cells were rinsed with PBS and stained with Nile red solution (Solarbio) at room temperature in the dark for 10 min. Excess stain was washed away with PBS. Nuclei were stained with 4',6-diamidino-2-phenylindole (DAPI) (Sigma-Aldrich) in PBS at room temperature in the dark for 5 min. After washing twice with PBS, cells were observed and photographed under a fluorescence microscope (Leica, Wetzlar, Germany). Nile red fluorescence was observed at excitation wavelength 543 nm and emission wavelength 598 nm, while DAPI fluorescence was observed at excitation wavelength 358 nm and emission wavelength 461 nm.

### Enzyme-linked immunosorbent assay

To quantify the protein levels of inflammatory cytokines (TNF-α, IL-6, and IL-1β), ELISA was performed. ELISA kits for TNF-α, IL-6, and IL-1β were purchased from JONLNBIO (Shanghai, China). The assays were conducted according to the manufacturer’s instructions. Cell culture supernatants and liver tissue homogenates from mice were collected and centrifuged at 1,500 *g* for 10 min at 4°C to remove debris. The supernatants were then stored at −80°C until analysis. Liver tissue samples were homogenized in PBS containing protease inhibitors (Roche), and the homogenates were centrifuged at 10,000 *g* for 15 min at 4°C, with the supernatants collected for ELISA.

The ELISA procedure involved coating ELISA plates with capture antibodies and incubating them overnight at 4°C. Plates were washed with PBS with 0.05% Tween-20 and blocked with 5% BSA in PBS for 1 h at room temperature. Samples and standards were added and incubated for 2 h at room temperature. After washing, detection antibodies were added and incubated for 1 h, followed by the addition of streptavidin-HRP. Absorbance was measured at 450 nm using a microplate reader (Bio-Rad, Hercules, CA).

### RNA-seq and bioinformatics analysis

Total RNA was extracted from treated HepG2 cells, and RNA-seq library construction and sequencing were performed by a professional service company (Novogene, Beijing, China). The library was constructed using the Illumina TruSeq RNA Sample Preparation Kit according to the manufacturer's instructions. Sequencing was conducted on the Illumina HiSeq platform, generating 150 bp paired-end reads. The sequencing data underwent quality control, with read quality assessed using FastQC software (Babraham Bioinformatics, Cambridge). Low-quality reads and adapter sequences were removed using Trimmomatic software (Usadel Lab, Aachen, Germany). The cleaned high-quality reads were aligned to the reference genome (e.g., human reference genome GRCh38) using HISAT2 (Johns Hopkins University, Baltimore, MD). Alignment results were used for transcript assembly and quantification analysis with StringTie software (Johns Hopkins University, Baltimore, MD). Differentially expressed gene analysis was conducted using the DESeq2 package (Bioconductor, Boston, MA) with thresholds set at |log2 fold change| > 1 and *P* value <0.05 to identify significantly differentially expressed genes. For these genes, gene ontology (GO) functional annotation and Kyoto Encyclopedia of Genes and Genomes pathway enrichment analysis were performed using ClusterProfiler (Bioconductor, Boston, MA).

For bioinformatics analysis of the GSE167523 dataset, the following steps were performed: GEO2R, an online platform provided by the Gene Expression Omnibus, was utilized for differential gene expression analysis of the GSE167523 dataset. This tool facilitated group comparisons to identify significantly upregulated and downregulated genes. The R package ggplot2 was employed to create volcano plots, visually representing the distribution of significantly differentially expressed genes based on their log2 fold change and *P* values. To visualize the expression patterns of significant genes across different samples, the pheatmap package in R was applied, enabling hierarchical clustering and heatmap generation.

### Chromatin immunoprecipitation

Chromatin immunoprecipitation (ChIP) was used to verify the binding of MTF1 to the MT1B promoter. According to the ChIP kit instructions (Millipore), cells were fixed with 1% formaldehyde at room temperature for 10 min to crosslink proteins and DNA, and the reaction was terminated by adding glycine (Sigma-Aldrich). Fixed cells were washed twice with PBS and lysed with cell lysis buffer. The lysates were sonicated to shear chromatin into 200–1,000 bp DNA fragments. The sheared chromatin was immunoprecipitated with an anti-MTF1 antibody or normal rabbit IgG as a control. Immunocomplexes were captured using protein A/G magnetic beads and incubated at room temperature for 2 h. Immunoprecipitated material was separated using a magnetic rack. The purified DNA was analyzed by quantitative polymerase chain reaction to assess the enrichment of the MT1B promoter region.

### Luciferase reporter assay

To verify the interaction between MTF1 and the MT1B promoter regions E1 and E2, a luciferase reporter assay was performed. A luciferase reporter vector containing the MT1B promoter region, pGL3-MT1B (Promega, Madison, WI), was constructed. The reporter vector and Renilla internal control vector pRL-TK (Promega) were cotransfected into HepG2 or AML12 cells using Lipofectamine 2,000. Forty-eight hours post-transfection, luciferase activity was detected using the Dual-Luciferase Reporter Assay System (Promega). Cell lysates were mixed with luciferase reagent, and luminescence signals were read using a microplate reader (Thermo Fisher Scientific). Luciferase activity was expressed as the ratio of Firefly luciferase to Renilla luciferase and normalized to the control group.

### Immunofluorescence

HepG2 and AML12 cells were seeded on coverslips and fixed with 4% paraformaldehyde (Sigma-Aldrich) for 30 min when cells reached 70%–80% confluency. After fixation, cells were permeabilized with 0.1% Triton X-100 (Solarbio) at room temperature for 10 min and blocked with 5% BSA (Solarbio) at room temperature for 1 h. After blocking, cells were incubated with the primary antibody against MTF1 at 4°C overnight. The next day, cells were washed three times with PBS, 5 min each, and then incubated with a fluorescently labeled secondary antibody at room temperature in the dark for 1 h. After secondary antibody incubation, cells were washed three times with PBS, 5 min each. Nuclei were stained with DAPI at room temperature in the dark for 5 min. After staining, cells were washed twice with PBS and mounted using an antifade mounting medium (Beyotime). Finally, cells were observed under a fluorescence microscope (Leica).

### Animal model construction

Six-week-old male C57BL/6 mice (SPF grade) were randomly assigned to each group, with six mice per group. The control group was given a normal diet, while the model group was fed a high-fat diet (HFD) for 8 and 16 weeks to induce MAFLD and MASH, respectively. The HFD was custom-formulated by Jiangsu Xietong Pharmaceutical Bio-engineering Co., Ltd. (Product No. XTHF60). The diet composition included approximately 35% fat, 26% protein, and 26% carbohydrates. For the in vivo MT1B knockdown group, recombinant adeno-associated virus (AAV8-shPNPT1) was administered via tail vein injection at a dose of 1 × 10^12^ genome copies per mice, using the vector element GV698 pAAV-ApoE/hAATp-EGFP-MIR155(MCS)-SV40 PolyA (Genechem, Shanghai, China) at week 8. During the tail vein injection of AAV8, all mice were under isoflurane anesthesia. In addition, MK-2206 was administered intraperitoneally 3 times a week at a dose of 10 mg/kg. The dose of ZnSO_4_ was 5 mg per kg body weight, administered via intraperitoneal injection (ip) three times a week for 8 weeks, starting from the eighth week, similar to MK-2206. At the end of the experiment, the mice were anesthetized, blood samples were collected, and the mice were euthanized to collect liver tissues. The liver tissues were used for the detection of TG, TC, and inflammatory factors (TNF-α, IL-6, IL-1β, and IL-10), and for Oil red O staining, Sirius Red staining, and Masson’s trichrome staining to assess steatosis and fibrosis levels. The Ethics Committee of The Second Affiliated Hospital of Harbin Medical University approved all animal experiments (YJSDW2023-069).

### Tissue section preparation and staining

To observe the degree of steatosis and fibrosis in liver tissues, tissue section preparation and staining were performed. Liver tissues were taken from control and treated mice. A portion of the liver tissue was quickly placed in OCT (Sakura Finetek, Torrance) and frozen. Frozen sections of 10 μm thickness were prepared using a cryostat (Leica), stained with Oil red O for 15 min, washed with 60% isopropanol buffer, counterstained with Harris hematoxylin (Solarbio) for 1 min, rinsed with PBS, and mounted. The remaining tissues were immediately fixed in 4% paraformaldehyde (Sigma-Aldrich) for 24 h. The fixed tissues were dehydrated with a series of ethanol solutions, cleared with xylene, and embedded in paraffin. Paraffin-embedded tissues were sectioned into 5 μm thick slices for tissue section preparation.

H&E staining was performed as follows: sections were baked at 60°C for 30 min, deparaffinized, and immersed in running water. They were then stained with hematoxylin (Solarbio) for 5 min, washed with running water for 10 min, differentiated in 1% hydrochloric acid ethanol for 10 s, washed with running water, blued in 0.5% ammonia water, washed with running water, stained with eosin (Solarbio) for 2 min, washed with running water, dehydrated through an ethanol gradient, cleared with xylene, and mounted. Sirius Red and Masson’s trichrome staining kits (Solarbio) were used to detect fibrosis levels in liver tissues. Briefly, after deparaffinization and hydration, the sections were stained according to the respective protocols, dehydrated with anhydrous ethanol, cleared with xylene, and mounted with neutral balsam.

### Statistical analysis

All experimental data were statistically analyzed using GraphPad Prism 8.0 (GraphPad Software, San Diego, CA). Data were expressed as mean ± SEM. Group comparisons were performed using two-tailed *t*-tests or one-way *ANOVA*, depending on the characteristics of the data. When significant differences were detected by *ANOVA*, Tukey's Honest Significant Difference test was conducted as the posthoc analysis to compare multiple groups. For non-normally distributed data, the *Mann–Whitney U* test or *Kruskal–Wallis* test was used. To ensure data reliability, each experiment was repeated at least three times. Normality and homogeneity of variance tests were conducted before statistical analysis, and a *P* value <0.05 was considered statistically significant. Significance levels were indicated as follows: ∗*P* < 0.05, ∗∗*P* < 0.01, and ∗∗∗*P* < 0.001.

## Results

### MT1B expression was downregulated in MASH tissues and FFA-induced cells

**Fig. 1:**
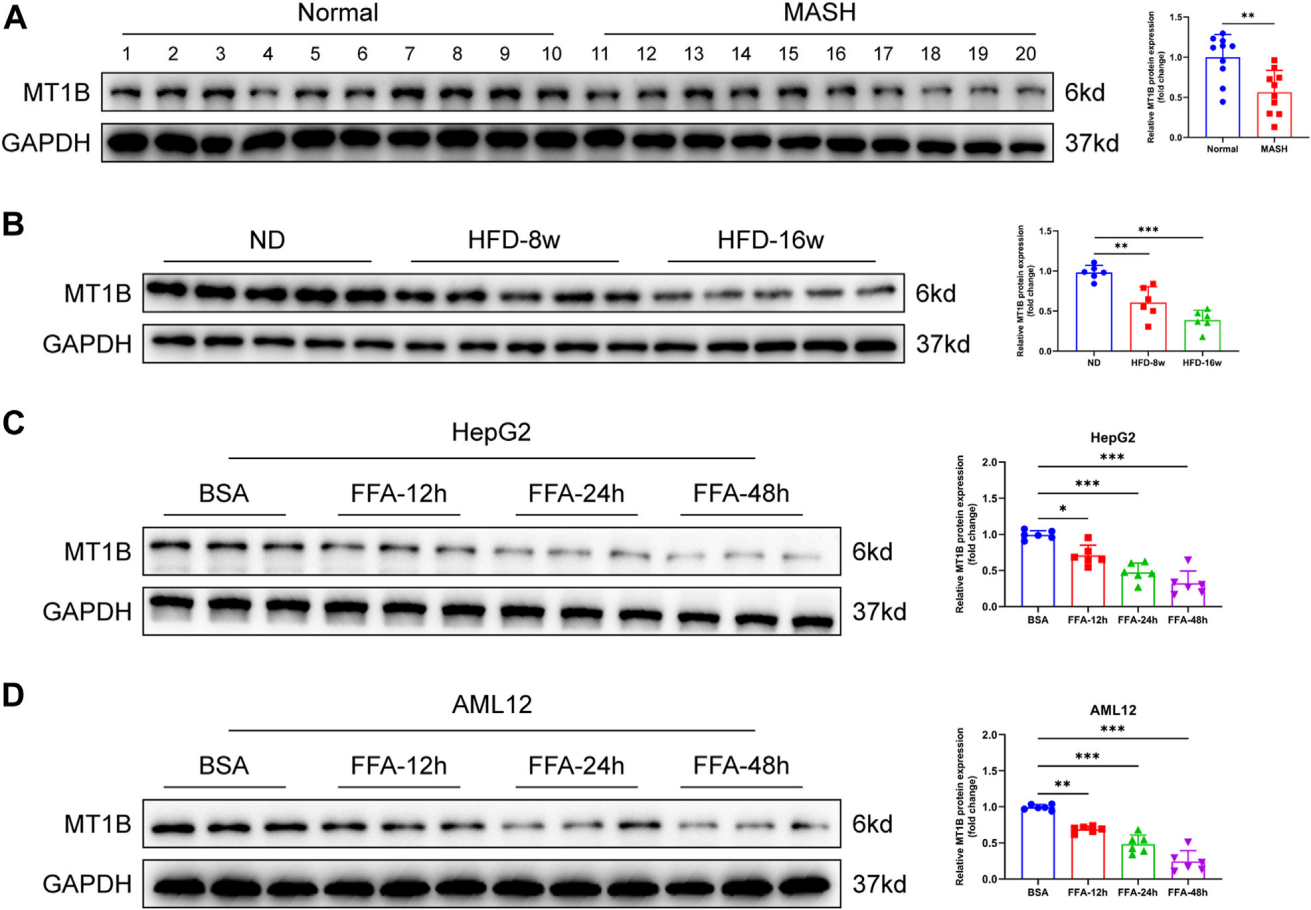
Downregulation of MT1B expression in MASH tissues and FFA-induced cells. A: Western blot analysis showed that MT1B protein expression levels were significantly lower in liver tissues from MASH patients than normal liver tissues (n = 10). B: In high-fat diet–induced mouse models, MT1B expression significantly decreased as MASH progressed (n = 6). C and D: In vitro FFA induction experiments showed that MT1B protein expression levels in HepG2 and AML12 cells gradually decreased with increasing induction time (n = 6). Protein levels were normalized to GAPDH. Data are represented as mean ± SD. Data in (A) were presented by two-tailed Student's *t* test. Data in (B–D) were presented by one-way ANOVA test. ∗*P* < 0.05, ∗∗*P* < 0.01, and ∗∗∗*P* < 0.001. MT1B, metallothionein 1B; MASH, metabolic dysfunction–associated steatohepatitis.

To identify potential key genes in MASH, we performed a bioinformatics analysis using the GSE167523 dataset. Volcano plots and heatmaps revealed that *MT1B* expression was significantly lower in MASH tissue samples than normal tissues (Supplementary Fig. S1A, B). We then further evaluated MT1B expression in tissues from MASH patients and in HFD-induced mouse models. qRT-PCR and Western blot analyses demonstrated that MT1B expression levels were significantly reduced in MASH patient tissues compared to normal liver tissues (Fig. 1A and Supplementary Fig. S1C). Similarly, in HFD-induced mouse models, MT1B expression exhibited a progressive downregulation as MASH severity increased (Fig. 1B and Supplementary Fig. S1D). To further investigate this phenomenon in vitro, we conducted high-fat induction experiments using FFAs (a mixture of palmitic acid and oleic acid) in human liver cells (HepG2) and mouse liver cells (AML12). The results showed that MT1B mRNA and protein expression levels in both HepG2 and AML12 cells were significantly decreased following FFA induction, with a gradual decline observed as the induction time increased, compared to the normal control group (Fig. 1C, D and Supplementary Fig. S1E, F). In summary, these results indicated that MT1B expression was markedly downregulated in both HFD-induced MASH tissues and FFA-induced cells, suggesting that MT1B may play a critical role in the pathogenesis of MASH.

### MT1B regulates lipid metabolism and inflammation in cells

**Fig. 2:**
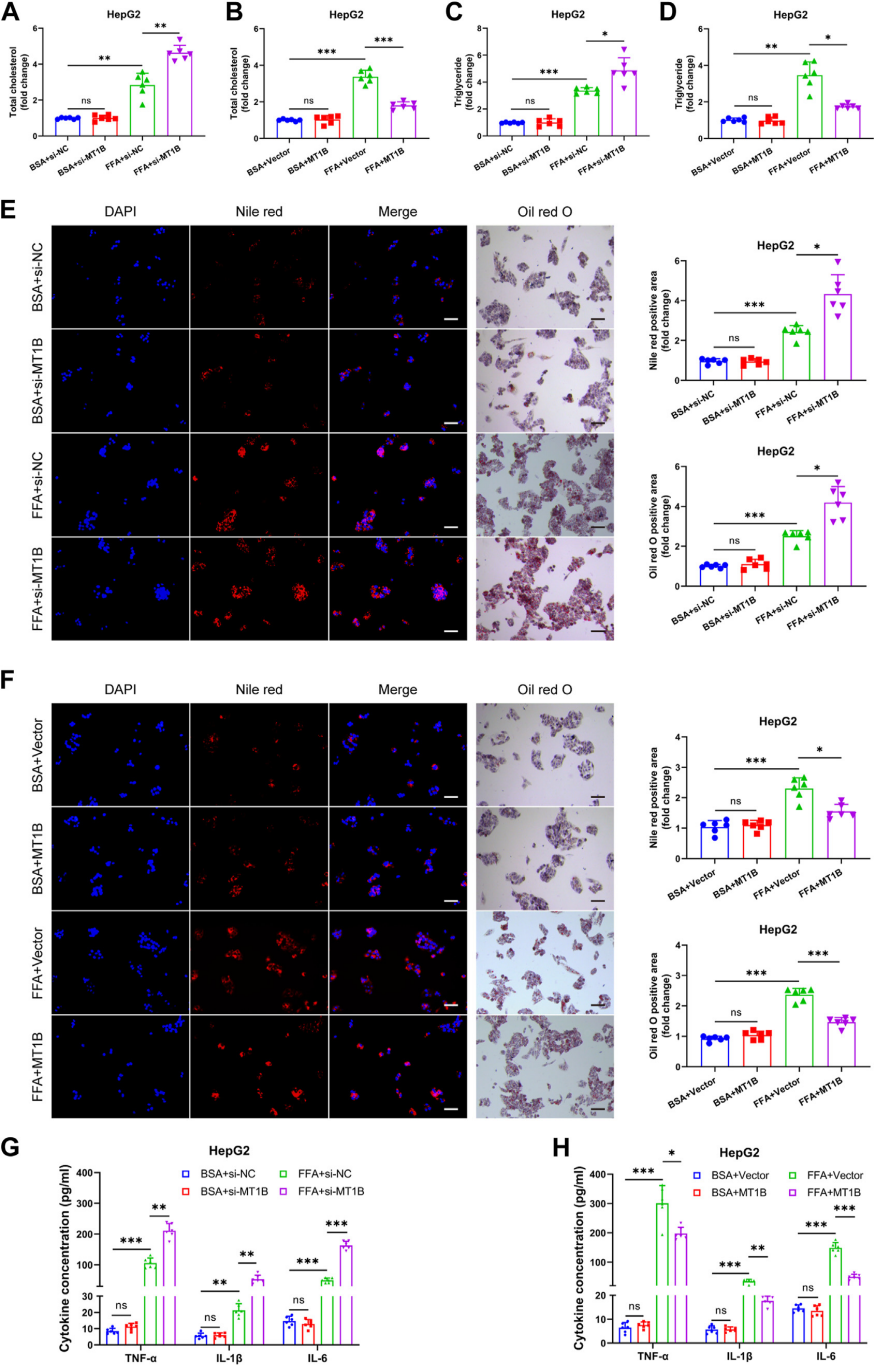
Regulation of lipid metabolism and inflammation by MT1B in HepG2 cells. A and B: Triglyceride (TG) levels increased in MT1B-knockdown and decreased in MT1B-overexpressing HepG2 cells (n = 6). C and D: Total cholesterol (TC) levels increased in MT1B-knockdown and decreased in MT1B-overexpressing HepG2 cells (n = 6). E and F: Nile red and Oil red O staining showed that the number and size of lipid droplets significantly increased in MT1B-knockdown cells and decreased in MT1B-overexpressing cells (n = 6). G: ELISA results showed that the expression of proinflammatory factors (TNF-α, IL-6, and IL-1β) was significantly upregulated in MT1B-knockdown HepG2 cells (n = 6). H: The expression of proinflammatory factors was significantly downregulated and the anti-inflammatory factor increased in MT1B-overexpressing HepG2 cells (n = 6). Data are represented as mean ± SD. Data in (A–F) were presented by one-way ANOVA test. Data in (G, H) were presented by two-way ANOVA test. ∗*P* < 0.05, ∗∗*P* < 0.01, and ∗∗∗*P* < 0.001. MT1B, metallothionein 1B.

**Fig. 3:**
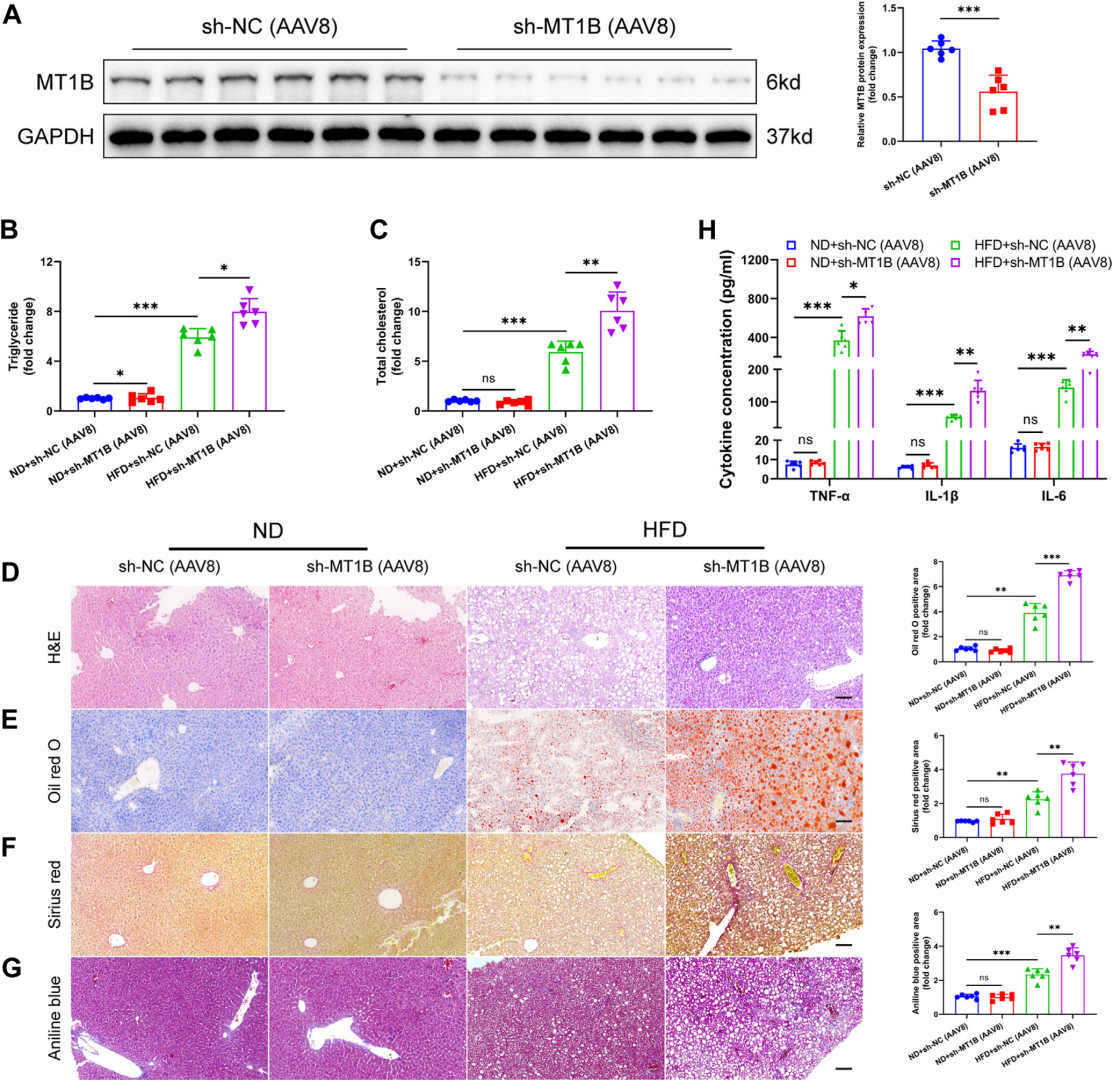
Specific downregulation of MT1B in mouse liver and its effects on lipid metabolism and inflammation. A: AAV8-shMT1B injection significantly downregulated MT1B expression in mouse liver (n = 6). B and C: Silencing MT1B significantly increased TC and TG levels in mouse liver (n = 6). D and E: Silencing MT1B significantly increased lipid accumulation in mouse liver (n = 6). F and G: Sirius Red and Masson’s trichrome staining showed that fibrosis significantly worsened in MT1B-knockdown mouse liver (n = 6). H: In MT1B-knockdown mouse liver, the expression of pro-inflammatory factors (TNF-α, IL-6, and IL-1β) was significantly upregulated by ELISA (n = 6). Data are represented as mean ± SD. Data in (A) were presented by two-tailed Student's *t* test. Data in (B–G) were presented by one-way ANOVA test. Data in (H) were presented by two-way ANOVA test. ∗*P* < 0.05, ∗∗*P* < 0.01, and ∗∗∗*P* < 0.001. AAV, adeno-associated virus; MT1B, metallothionein 1B.

This study further investigated the effects of MT1B on lipid metabolism. First, we successfully achieved significant downregulation or upregulation of *MT1B* in HepG2 and AML12 cell by transfecting them with exogenous *MT1B* siRNA or overexpression vectors (Supplementary Fig. S2A–H). Quantification of TC and TG levels revealed that *MT1B* silencing resulted in a marked increase in both TG and TC (Fig. 2A, B and Supplementary Fig. S3A, B). Conversely, cells overexpressing *MT1B* showed reduced lipid droplet formation and significantly lower TG and TC levels (Fig. 2C, D and Supplementary Fig. S3C, D). Subsequent Oil red O and Nile red staining experiments further revealed that the number and size of lipid droplets were significantly increased in *MT1B*-silenced cells (Fig. 2E, F and Supplementary Fig. S3E, F). Additionally, downregulation of *MT1B* was associated with the upregulation of proinflammatory factors such as TNF-α, IL-6, and IL-1β (Fig. 2G and Supplementary Fig. S3G). Conversely, *MT1B* overexpression led to a reduction in the expression levels of these proinflammatory cytokines (Fig. 2H and Supplementary Fig. S3H). To further validate these findings in vivo, we specifically downregulated Mt1b expression in mouse livers using AAV8-shMT1B injection (Fig. 3A). The results showed that silencing Mt1b significantly increased TG and TC levels, accompanied by enhanced lipid accumulation in the liver (Fig. 3B–E). Furthermore, reduced Mt1b expression aggravated liver fibrosis (Fig. 3F, G) and led to the upregulation of proinflammatory factors in the liver (Fig. 3H). In summary, our findings indicate that MT1B plays a crucial role in lipid metabolism and inflammatory response.

### MT1B upregulation inhibited the activation of AKT/PI3K pathway in MASH

**Fig. 4:**
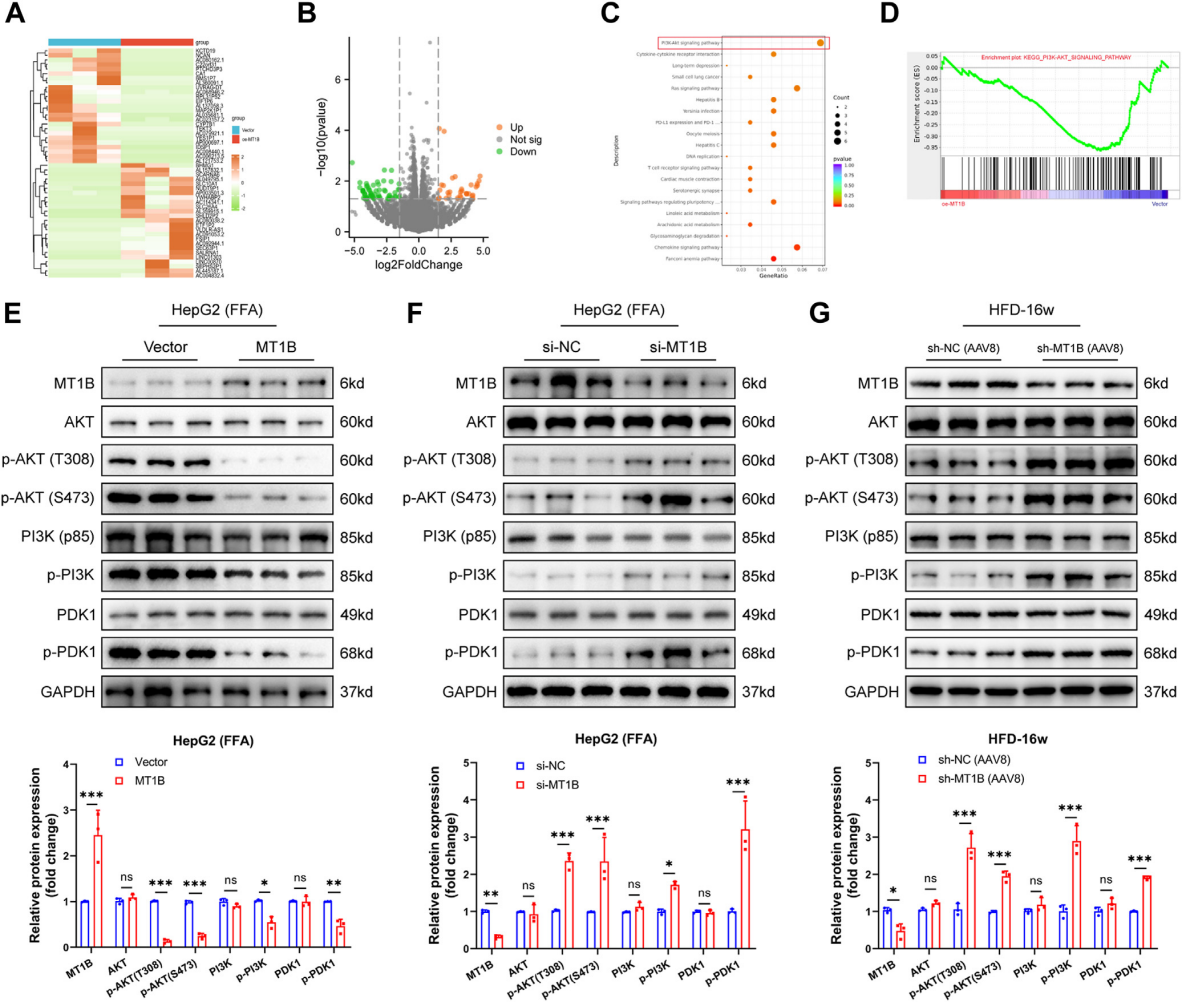
Upregulation of MT1B inhibits activation of AKT/PI3K signaling pathway in MASH. A and B: Heatmap and volcano plot of differentially expressed genes identified by RNA-seq in MT1B-overexpressing HepG2 cells. C and D: Differentially expressed genes were used for KEGG pathway enrichment and GSEA analysis of the AKT/PI3K signaling pathways. E: Western blot analysis showed that in MT1B-overexpressing cells, there were no significant changes in the expression of AKT, PI3K, and PDK1, but the expression of p-AKT, p-PI3K, and p-PDK1 significantly decreased (n = 3). F: In MT1B-silenced cells, the expression of p-AKT, p-PI3K, and p-PDK1 significantly increased (n = 3). G: The expression of p-AKT, p-PI3K, and p-PDK1 increased significantly after MT1B knockdown in mouse liver (n = 3). Phosphorylated protein levels (e.g., p-AKT, p-PI3K, and p-PDK1) were normalized to their respective total protein levels (e.g., AKT, PI3K, and PDK1) to account for variations in total protein expression. Data are represented as mean ± SD. Data in (E–G) were presented by two-way ANOVA test. ∗*P* < 0.05, ∗∗*P* < 0.01, and ∗∗∗*P* < 0.001. KEGG, Kyoto Encyclopedia of Genes and Genomes; MT1B, metallothionein 1B; MASH, metabolic dysfunction–associated steatohepatitis.

In further exploration of the mechanisms underlying MT1B’s protective role in MASH, RNA-seq and bioinformatics analyses of cells overexpressing *MT1B* revealed significant enrichment of the AKT/PI3K signaling pathway (Fig. 4A–D). The PI3K/AKT pathway is critical in MASH as it mediates various metabolic and inflammatory processes (31, 32, 33). Activation of this pathway has been shown to exacerbate inflammation, leading to more severe liver pathology (34). Deficiency in the gluconeogenic enzyme PCK1 exacerbates MAFLD through activation of the PI3K/AKT/platelet-derived growth factor axis (35). In this study, Western blot analysis showed that upregulation of MT1B did not significantly change the expression of AKT, PI3K, and PDK1, but markedly reduced the expression of phosphorylated AKT (p-AKT), phosphorylated PI3K (p-PI3K), and phosphorylated PDK1 (p-PDK1) (Fig. 4E). Conversely, in vitro and in vivo experiments had shown that silencing Mt1b led to opposite effects (Fig. 4F, G). These results suggest that MT1B can inhibit the activation of the AKT/PI3K pathway in MASH.

### Inhibition of AKT reversed the effects of MT1B silencing on MASH

**Fig. 5:**
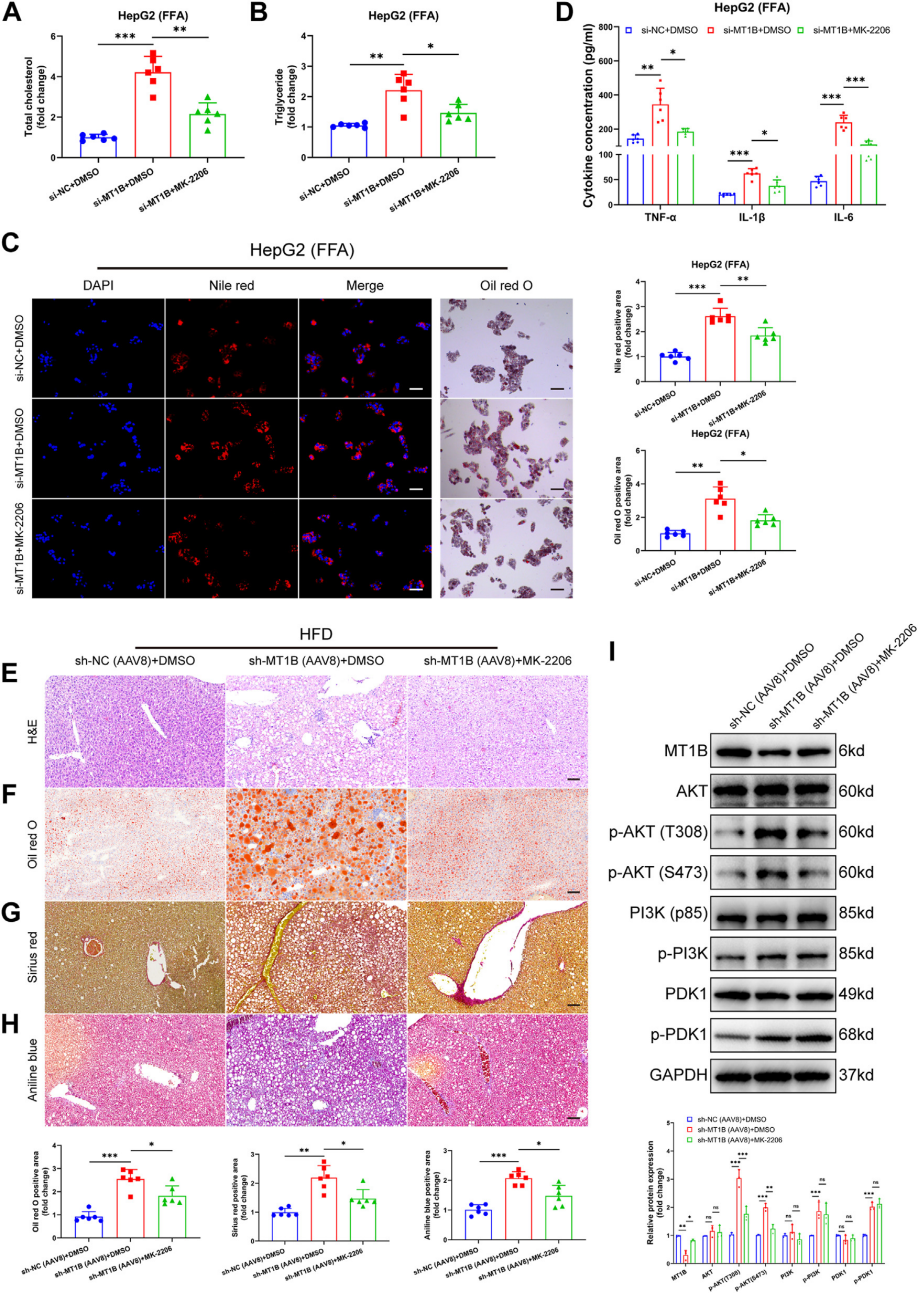
AKT inhibitor reverses the effects of MT1B silencing on MASH. A and B: Quantitative analysis of TC and TG shows that the AKT inhibitor (MK-2206) reversed the increase in TC and TG levels caused by MT1B silencing in HepG2 cells (n = 6). C: Oil red O and Nile red staining indicate that MK-2206 reversed the increased lipid accumulation in MT1B-silenced HepG2 cells (n = 6). D: The AKT inhibitor reversed the upregulation of proinflammatory factors (TNF-α, IL-6, and IL-1β) caused by MT1B silencing (n = 6). E and F: In MT1B-silenced mouse livers, the AKT inhibitor significantly reduced lipid accumulation induced by MT1B downregulation (n = 6). G and H: Sirius Red and Masson’s trichrome staining showed that the AKT inhibitor reversed the exacerbation of liver fibrosis caused by MT1B silencing in mice (n = 6). I: Western blot results showed that MK-2206 reversed increased p-AKT (T308) and p-AKT (S473) in mouse liver tissue after MT1B silencing (n = 3). Data are represented as mean ± SD. Data in (A–C and F–H) were presented by one-way ANOVA test. Data in (D and I) were presented by two-way ANOVA test. ∗*P* < 0.05, ∗∗*P* < 0.01, and ∗∗∗*P* < 0.001. MT1B, metallothionein 1B; TC, total cholesterol; TG, triglyceride.

To further elucidate the regulatory relationship between MT1B and the AKT/PI3K signaling pathway in MASH, we performed Oil red O and Nile red staining experiments and measured TG and TC levels. The results showed that the exacerbation of lipid metabolic disorders caused by *MT1B* silencing in HepG2 and AML12 cells could be rescued by treatment with AKT inhibitor (Fig. 5A–C and Supplementary Fig. S4A–C). Additionally, AKT inhibition reversed the increase in inflammatory factor expression induced by *MT1B* downregulation (Fig. 5D and Supplementary Fig. S4D). In vivo, specific downregulation of Mt1b in mouse liver significantly increased TG and TC levels and lipid accumulation (Supplementary Fig. S4E, F and Fig. 5E, F), aggravated liver fibrosis (Fig. 5G, H), and upregulated proinflammatory factors (Supplementary Fig. S4G), along with an increase in p-AKT expression (Fig. 5I). Treatment with AKT inhibitor mitigated these effects. In summary, our results indicate that MT1B influences the progression of MASH by regulating the AKT/PI3K signaling pathway.

### Zinc promoted the binding of MTF1 to MT1B, thus mitigating the progression of MASH

**Fig. 6:**
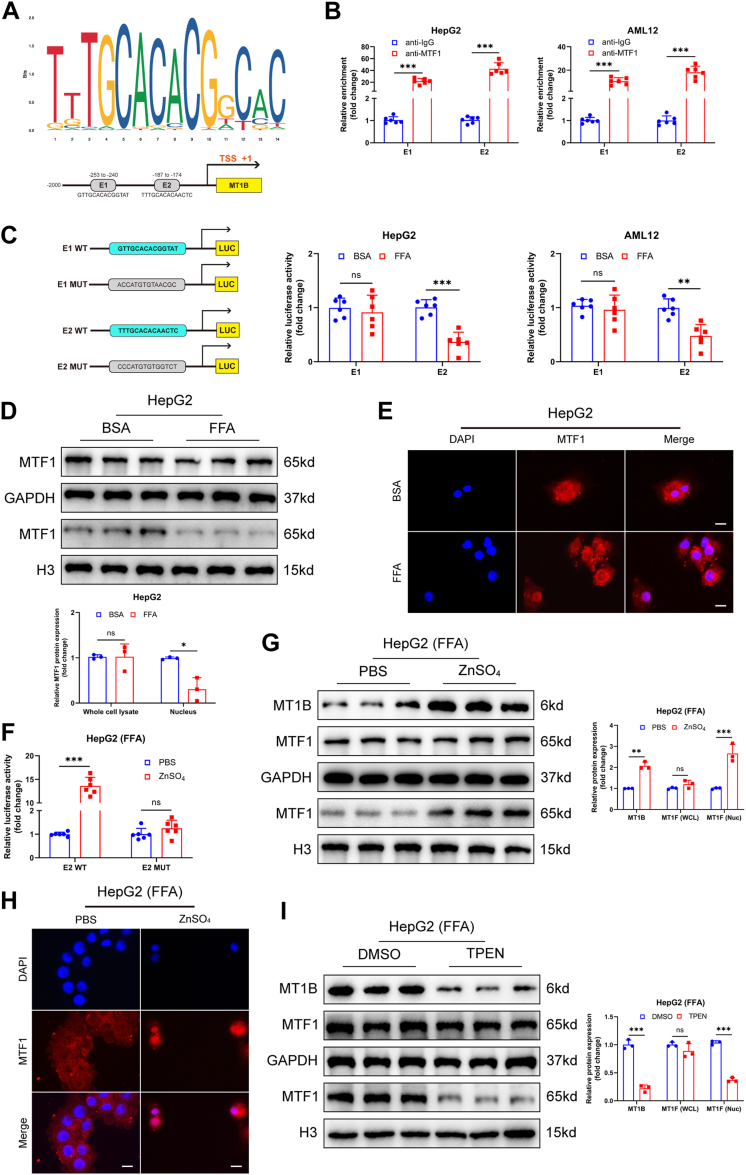
Zn^2+^ promote the binding of MTF1 to MT1B and mitigate MASH progression. A: JASPAR database prediction indicates that MTF1 has three potential binding sites (E1, E2, and E3) in the MT1B promoter region. B: ChIP assay confirmed that MTF1 primarily binds to the E1 and E2 sites of the MT1B promoter (n = 6). C: Luciferase reporter assay showed that MTF1 significantly enhanced the activity of the MT1B promoter at the E2 site (n = 6). D: Under FFA treatment, the total protein level of MTF1 did not change significantly, but its level in the nucleus was markedly reduced (n = 3). E: Immunofluorescence results showed that FFA treatment significantly reduced the nuclear translocation of MTF1. F: Luciferase reporter assay indicated that Zn^2+^ treatment enhanced the binding activity of MTF1 to the MT1B promoter (n = 6). G and H: Western blot and immunofluorescence results demonstrated that Zn^2+^ treatment significantly increased the nuclear translocation of MTF1 (n = 3). I: Western blot results demonstrate that Zn^2+^ inhibitor (TPEN) significantly inhibited the nuclear translocation of MTF1 in HepG2 cells (n = 3). Data are represented as mean ± SD. Data in (B–D, F, G, and I) were presented by two-way ANOVA test. ∗∗*P* < 0.01 and ∗∗∗*P* < 0.001. ChIP, chromatin immunoprecipitation; MT1B, metallothionein 1B; MASH, metabolic dysfunction–associated steatohepatitis.

**Fig. 7:**
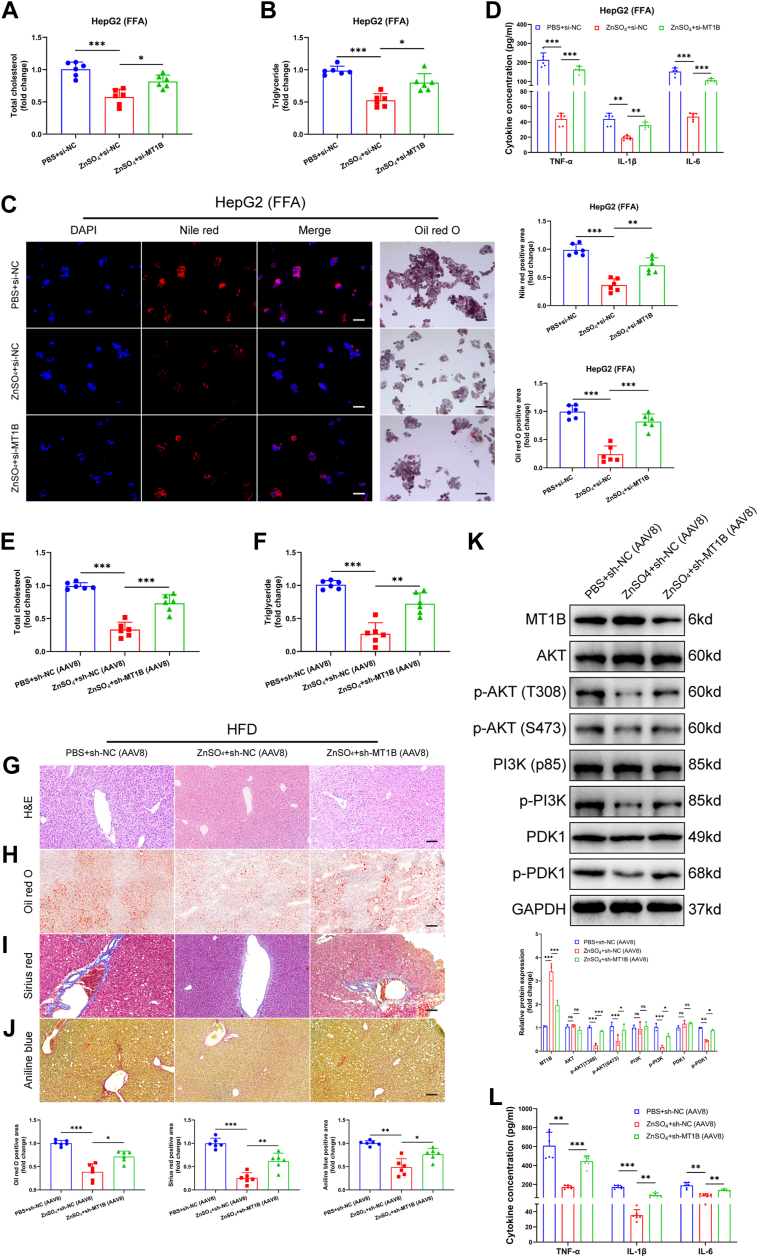
Zn^2+^ alleviate high-fat–induced lipid metabolism disorder and inflammation by upregulating MT1B. A–D: In HepG2 cells, Zn^2+^ treatment significantly alleviated FFA-induced lipid metabolism disorders and inflammatory factor expression, but MT1B silencing reversed these effects (n = 6). E–H: In HFD-induced mouse liver tissues, Zn^2+^ feeding significantly reduced lipid accumulation, which was reversed by MT1B downregulation (n = 6). I and J: Sirius Red and Masson’s trichrome staining showed that MT1B downregulation reversed the alleviation of liver fibrosis induced by Zn^2+^ treatment in mice (n = 6). K: Western blot results showed that Zn^2+^ treatment significantly inhibited the expression of p-AKT, p-PI3K, and p-PDK1, while MT1B silencing reversed the activation of AKT/PI3K signaling pathway by Zn^2+^ (n = 3). L: ELISA results showed that Zn^2+^ treatment significantly reduced the expression of proinflammatory factors (TNF-α, IL-6, and IL-1β), but MT1B silencing reversed the anti-inflammatory effects of Zn^2+^ (n = 6). Data are represented as mean ± SD. Data in (A–C and E–J) were presented by one-way ANOVA test. Data in (D, K, and L) were presented by two-way ANOVA test. ∗*P* < 0.05, ∗∗*P* < 0.01, and ∗∗∗*P* < 0.001. MT1B, metallothionein 1B; HFD, high-fat diet.

To further investigate the upstream molecular mechanisms regulating MT1B expression, we hypothesized that specific transcription factors might be involved, given the abnormal downregulation of MT1B mRNA in MASH. Previous studies have shown that MTF1 can activate the transcription of the MT family genes (36, 37), but its role in regulating MT1B remains unclear. Using JASPAR to predict the binding sites of *MT1B* and the transcription factor MTF1, we found three potential binding sites (E1, E2, and E3) in the *MT1B* promoter region (Fig. 6A). ChIP and luciferase reporter assays confirmed that MTF1 primarily regulated *MT1B* expression by binding to the E1 site (Fig. 6B, C). Furthermore, while the total MTF1 protein levels were not affected by FFA treatment, the nuclear MTF1 protein levels were significantly lower than the control group (Fig. 6D and Supplementary Fig. S5A). Immunofluorescence also showed that the nuclear translocation of MTF1 was significantly reduced under FFA conditions (Fig. 6E and Supplementary Fig. S5B). Previous studies have indicated that MTF1 is a metal-responsive transcription factor, particularly sensitive to Zn^2+^ concentration changes (37, 38). Luciferase reporter assays confirmed that Zn^2+^ enhanced the binding of MTF1 to the *MT1B* promoter (Fig. 6F and Supplementary Fig. S5C). Further immunofluorescence and Western blot experiments demonstrated that zinc sulfate treatment increased the nuclear translocation of MTF1 under FFA conditions (Fig. 6G, H and Supplementary Fig. S5D, E). In addition, the nuclear translocation of MTF1 was inhibited by the use of Zn ion inhibitor (TPEN) (Fig. 6I and Supplementary Fig. S5F). Further rescue experiments confirmed that Zn^2+^ could alleviate high-fat–induced lipid metabolic disorders, inhibit the activation of AKT/PI3K pathway and the expression of inflammatory factors in vitro and in vivo, but these effects were reversed by Mt1b silencing (Fig. 7A–L; Supplementary Fig. S6A–D). Moreover, after the application of TPEN chelating Zn^2+^, TC and TG levels were significantly increased, lipid droplet accumulation was increased, and the expression of inflammatory factors was significantly upregulated. However, overexpression of MT1B could reverse these conditions in FFA-induced HepG2 and AML12 cells (Supplementary Fig. S7A–H), respectively. These results indicate that Zn^2+^ promoted the nuclear translocation of MTF1, thereby upregulating MT1B expression and ultimately mitigating the progression of MASH.

## Discussion

MASH is a complex liver disease characterized by hepatic steatosis, inflammation, and fibrosis, which can eventually lead to cirrhosis and hepatocellular carcinoma (39, 40, 41, 42). In contrast to simple steatosis, MASH pathogenesis involves intricate metabolic pathways and inflammatory responses (43, 44, 45, 46). Given the significant prevalence and pathogenic potential of MASH, understanding its molecular mechanisms is crucial for developing effective therapeutic strategies. Recent studies have indicated that the expression changes of MT1B are closely associated with the progression of various liver diseases (21, 29, 30). However, the specific roles and regulatory mechanisms of MT1B in MASH remain unclear. Deciphering the function of MT1B in MASH could potentially provide new targets and strategies for MASH therapy.

In this study, we observed that *MT1B* expression was significantly downregulated in liver tissues of MASH patients and in HFD-induced mouse models, consistent with the analysis of the GSE167523 database using bioinformatics tools. The decreased expression of MT1B was closely associated with elevated TG and TC levels in hepatocytes, increased lipid droplet formation, and significantly upregulated proinflammatory factors. In vivo experiments, specific downregulation of Mt1b expression in mouse liver through AAV8-shMT1B injection significantly increased TG and TC cholesterol levels, exacerbated lipid accumulation, and led to significant upregulation of liver fibrosis and pro-inflammatory factors. These observations were consistent with in vitro findings, thereby highlighting the critical role of MT1B in suppressing inflammatory responses and maintaining lipid metabolic homeostasis.

To further elucidate the regulatory mechanisms of MT1B, we performed RNA-seq and bioinformatics analysis, which demonstrated that MT1B overexpression significantly inhibited the activation of the AKT/PI3K signaling pathway. Experimental results confirmed that MT1B upregulation resulted in reduced expression of phosphorylated AKT (p-AKT), PI3K (p-PI3K), and PDK1 (p-PDK1), while downregulation of MT1B led to the opposite effect. Moreover, the use of AKT inhibitors successfully reversed the lipid metabolic disturbances and inflammatory responses induced by MT1B downregulation, further supporting the pivotal role of the AKT/PI3K pathway in MT1B-mediated metabolic regulation. Additionally, our study found that Zinc could promote the binding of the transcription factor MTF1 to the Mt1b promoter, thereby upregulating Mt1b expression. Rescue experiments in vitro and in vivo confirmed that Zn ion treatment alleviate HFD-induced lipid metabolic disorders and inhibit the expression of inflammatory factors, but these effects were reversed by Mt1b silencing. These results suggest that Zn^2+^ promoted the nuclear translocation of MTF1, further upregulating MT1B expression, and ultimately mitigating the progression of MASH.

**Fig. 8:**
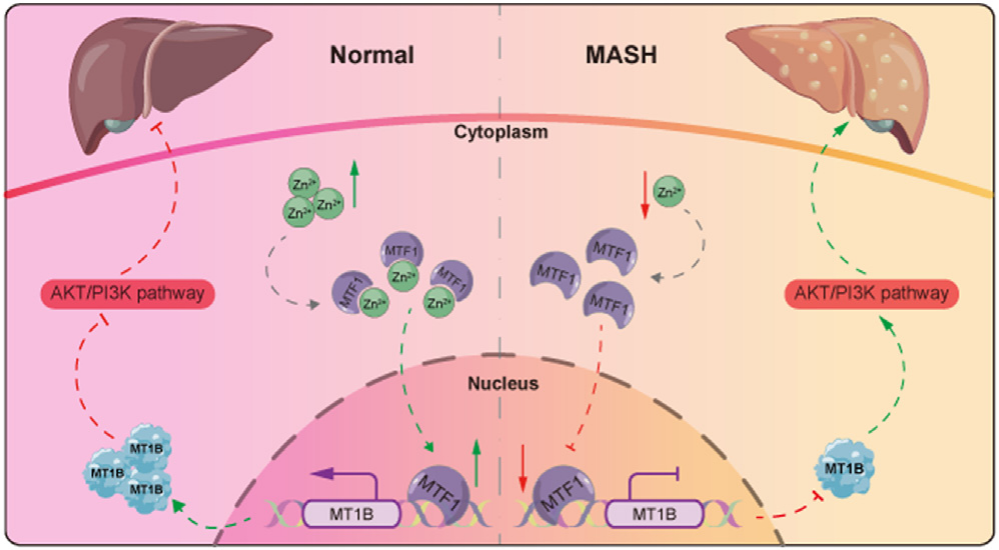
The schematic diagram of MT1B regulated by Zn^2+^ delaying MASH progression by inhibiting AKT/PI3K signaling pathway. Under normal conditions, intracellular Zn^2+^ in the cytoplasm facilitates the nuclear translocation of transcription factor MTF1, thereby upregulating MT1B expression and suppressing the AKT/PI3K pathway. In MASH, however, reduced nuclear translocation of MTF1 results in downregulated MT1B expression that promotes activation of the AKT/PI3K pathway, consequently exacerbating hepatic lipid metabolic disorder and increasing inflammatory factors. MT1B, metallothionein 1B; MASH, metabolic dysfunction–associated steatohepatitis.

Overall, this study provides crucial insights into the regulatory role of MT1B in MASH and offers a new perspective on the molecular mechanisms underlying the disease. First, our results establish MT1B as a key modulator of lipid metabolism and inflammatory responses through its inhibitory effect on the AKT/PI3K signaling pathway, identifying novel therapeutic targets for MASH treatment. Second, the regulatory role of Zinc through MTF1 and MT1B in MASH adds an additional layer of complexity to the disease's pathogenesis, suggesting potential avenues for therapeutic intervention (Fig. 8). Nevertheless, this study has several limitations. Although the role of MT1B in MASH was validated both in vitro and in vivo, further research is needed to investigate the long-term effects and behavior of MT1B in more complex physiological environments. Additionally, while we identified the AKT/PI3K pathway as a critical mediator of MT1B function, other unexplored signaling pathways may also be involved. Last, our research primarily focused on the expression and functional changes of MT1B, thus future research should investigate the broader gene regulatory network and explore potential pharmacological interventions targeting MT1B.

In conclusion, this study systematically revealed the crucial roles and regulatory mechanisms of MT1B in MASH through comprehensive experiments and analyses. These findings not only deepen our understanding of MASH pathogenesis but also provide theoretical and experimental support for developing new therapeutic strategies. Future research will focus on further exploring the biological functions of MT1B and its potential applications in other metabolic diseases, aiming to achieve breakthroughs in the diagnosis and treatment of related diseases.

## Data availability

The datasets that support the findings of this study are available in this article or the Supplemental Information.

## Supplemental data

This article contains supplemental data.

## Conflicts of interests

The authors declare that they have no conflicts of interest with the contents of this article.

## References

[1] De A., Bhagat N., Mehta M., Taneja S., Duseja A. (2024). Metabolic dysfunction-associated steatotic liver disease (MASLD) definition is better than MAFLD criteria for lean patients with NAFLD. J. Hepatol..

[2] Jiang Y., Luo P., Cao Y., Peng D., Huo S., Guo J. (2024). The role of STAT3/VAV3 in glucolipid metabolism during the development of HFD-induced MAFLD. Int. J. Biol. Sci..

[3] Barrow F., Khan S., Wang H., Revelo X.S. (2021). The emerging role of B cells in the pathogenesis of NAFLD. Hepatology.

[4] Guan C.H., Zou X.L., Yang C.R., Shi W.J., Gao J.J., Ge Y.F. (2024). Polyribonucleotide nucleotidyltransferase 1 participates in metabolic-associated fatty liver disease pathogenesis by affecting lipid metabolism and mitochondrial homeostasis. Mol. Metab..

[5] Schwarzler J., Grabherr F., Grander C., Adolph T.E., Tilg H. (2024). The pathophysiology of MASLD: an immunometabolic perspective. Expert Rev. Clin. Immunol..

[6] Hutchison A.L., Tavaglione F., Romeo S., Charlton M. (2023). Endocrine aspects of metabolic dysfunction-associated steatotic liver disease (MASLD): beyond insulin resistance. J. Hepatol..

[7] Targher G., Corey K.E., Byrne C.D., Roden M. (2021). The complex link between NAFLD and type 2 diabetes mellitus - mechanisms and treatments. Nat. Rev. Gastroenterol. Hepatol..

[8] Younossi Z.M., Golabi P., Paik J.M., Henry A., Van Dongen C., Henry L. (2023). The global epidemiology of nonalcoholic fatty liver disease (NAFLD) and nonalcoholic steatohepatitis (NASH): a systematic review. Hepatology.

[9] Lazarus J.V., Mark H.E., Villota-Rivas M., Palayew A., Carrieri P., Colombo M. (2022). The global NAFLD policy review and preparedness index: are countries ready to address this silent public health challenge?. J. Hepatol..

[10] Younossi Z., Tacke F., Arrese M., Chander Sharma B., Mostafa I., Bugianesi E. (2019). Global perspectives on nonalcoholic fatty liver disease and nonalcoholic steatohepatitis. Hepatology.

[11] Gopoju R., Wang J., Pan X., Hu S., Lin L., Clark A. (2024). Hepatic FOXA3 overexpression prevents Western diet-induced obesity and MASH through TGR5. J. Lipid Res..

[12] Wang X.L., Xie Q. (2022). Metabolic dysfunction-associated fatty liver disease (MAFLD) and viral hepatitis. J. Clin. Transl. Hepatol..

[13] Nunes J.R.C., O'Dwyer C., Ghorbani P., Smith T.K.T., Chauhan S., Robert-Gostlin V. (2024). Myeloid AMPK signaling restricts fibrosis but is not required for metformin improvements during CDAHFD-induced NASH in mice. J. Lipid Res..

[14] Portincasa P., Bonfrate L., Khalil M., De Angelis M., Calabrese F.M., D'Amato M. (2022). Intestinal barrier and permeability in health, obesity and NAFLD. Biomedicines.

[15] Yao X., Dong R., Hu S., Liu Z., Cui J., Hu F. (2023). Tripartite motif 38 alleviates the pathological process of NAFLD-NASH by promoting TAB2 degradation. J. Lipid Res..

[16] Ni Y., Zhuge F., Ni L., Nagata N., Yamashita T., Mukaida N. (2022). CX3CL1/CX3CR1 interaction protects against lipotoxicity-induced nonalcoholic steatohepatitis by regulating macrophage migration and M1/M2 status. Metabolism.

[17] Liu H., Li D., Sun L., Qin H., Fan A., Meng L. (2022). Interaction of lncRNA MIR100HG with hnRNPA2B1 facilitates m(6)A-dependent stabilization of TCF7L2 mRNA and colorectal cancer progression. Mol. Cancer.

[18] Moore M.P., Cunningham R.P., Meers G.M., Johnson S.A., Wheeler A.A., Ganga R.R. (2022). Compromised hepatic mitochondrial fatty acid oxidation and reduced markers of mitochondrial turnover in human NAFLD. Hepatology.

[19] Scorletti E., Carr R.M.M. (2022). A new perspective on NAFLD: focusing on lipid droplets. J. Hepatol..

[20] Aron-Wisnewsky J., Vigliotti C., Witjes J., Le P., Holleboom A.G., Verheij J. (2020). Gut microbiota and human NAFLD: disentangling microbial signatures from metabolic disorders. Nat. Rev. Gastroenterol. Hepatol..

[21] Dai H., Wang L., Li L., Huang Z., Ye L. (2021). Metallothionein 1: a new spotlight on inflammatory diseases. Front. Immunol..

[22] Luo J., Liu X., Zhang Y., Yin M., Xu L., Cao M. (2023). Coexpression network analysis identified MT3 as a hub gene that promotes the chemoresistance of oral cancer by regulating the expression of YAP1. BMC Oral Health.

[23] Wu J., Jia S., Xu B., Yao X., Shao J., Yao J. (2023). Bicyclol attenuates high fat diet-induced non-alcoholic fatty liver disease/non-alcoholic steatohepatitis through modulating multiple pathways in mice. Front. Pharmacol..

[24] Yan X., Cao N., Chen Y., Lan H.Y., Cha J.H., Yang W.H. (2020). MT4-MMP promotes invadopodia formation and cell motility in FaDu head and neck cancer cells. Biochem. Biophys. Res. Commun..

[25] Xue Z., Wu X., Chen X., Luo Q. (2016). MT3-MMP down-regulation promotes tumorigenesis and correlates to poor prognosis in esophageal squamous cell carcinoma. Cancer Med..

[26] Guan Q., Wang Z., Hu K., Cao J., Dong Y., Chen Y. (2023). Melatonin ameliorates hepatic ferroptosis in NAFLD by inhibiting ER stress via the MT2/cAMP/PKA/IRE1 signaling pathway. Int. J. Biol. Sci..

[27] Wang Y., Wang G., Tan X., Ke K., Zhao B., Cheng N. (2019). MT1G serves as a tumor suppressor in hepatocellular carcinoma by interacting with p53. Oncogenesis.

[28] Li X., Zhong S., Sun Y., Huang X., Li Y., Wang L. (2022). Integration analysis identifies the role of metallothionein in the progression from hepatic steatosis to steatohepatitis. Front. Endocrinol. (Lausanne).

[29] Ogushi S., Kimura T. (2022). The difference in zinc concentrations required for induction among metallothionein isoforms can be explained by the different MTF1 affinities to MREs in its promoter. Int. J. Mol. Sci..

[30] Si M., Lang J. (2018). The roles of metallothioneins in carcinogenesis. J. Hematol. Oncol..

[31] Bu L., Zhang Z., Chen J., Fan Y., Guo J., Su Y. (2024). High-fat diet promotes liver tumorigenesis via palmitoylation and activation of AKT. Gut.

[32] Xi Y., Kim S., Nguyen T.T.T., Lee P.J., Zheng J., Lin Z. (2023). 2-Geranyl-1-methoxyerythrabyssin II alleviates lipid accumulation and inflammation in hepatocytes through AMPK activation and AKT inhibition. Arch. Pharm. Res..

[33] Xu G., Fan L., Zhao S., OuYang C. (2022). MT1G inhibits the growth and epithelial-mesenchymal transition of gastric cancer cells by regulating the PI3K/AKT signaling pathway. Genet. Mol. Biol..

[34] Li J., Wang T., Liu P., Yang F., Wang X., Zheng W. (2021). Hesperetin ameliorates hepatic oxidative stress and inflammation via the PI3K/AKT-Nrf2-ARE pathway in oleic acid-induced HepG2 cells and a rat model of high-fat diet-induced NAFLD. Food Funct..

[35] Ye Q., Liu Y., Zhang G., Deng H., Wang X., Tuo L. (2023). Deficiency of gluconeogenic enzyme PCK1 promotes metabolic-associated fatty liver disease through PI3K/AKT/PDGF axis activation in male mice. Nat. Commun..

[36] Zhang B., Li M., Zhou G., Gu X., Xie L., Zhao M. (2023). ZnO-NPs alleviate aflatoxin B(1)-induced hepatoxicity in ducklings by promoting hepatic metallothionein expression. Ecotoxicol Environ. Saf..

[37] Grzywacz A., Gdula-Argasinska J., Muszynska B., Tyszka-Czochara M., Librowski T., Opoka W. (2015). Metal responsive transcription factor 1 (MTF-1) regulates zinc dependent cellular processes at the molecular level. Acta Biochim. Pol..

[38] Dong G., Chen H., Qi M., Dou Y., Wang Q. (2015). Balance between metallothionein and metal response element binding transcription factor 1 is mediated by zinc ions (review). Mol. Med. Rep..

[39] Huang Z., Xia H., Cui Y., Yam J.W.P., Xu Y. (2023). Ferroptosis: from basic research to clinical therapeutics in hepatocellular carcinoma. J. Clin. Transl. Hepatol..

[40] Yip T.C.F., Lee H.W., Chan W.K., Wong G.L.H., Wong V.W.S. (2022). Asian perspective on NAFLD-associated HCC. J. Hepatol..

[41] Neuendorf H.M., Simmons J.L., Boyle G.M. (2023). Therapeutic targeting of anoikis resistance in cutaneous melanoma metastasis. Front. Cell Dev. Biol..

[42] Liu Y., Zhao Y., Liu Q., Li B., Daniel P.V., Chen B. (2024). Effects of apolipoprotein H downregulation on lipid metabolism, fatty liver disease, and gut microbiota dysbiosis. J. Lipid Res..

[43] Kazankov K., Jorgensen S.M.D., Thomsen K.L., Moller H.J., Vilstrup H., George J. (2019). The role of macrophages in nonalcoholic fatty liver disease and nonalcoholic steatohepatitis. Nat. Rev. Gastroenterol. Hepatol..

[44] Shankar K., Metzger N.P., Lawrence C., Gupta D., Osborne-Lawrence S., Varshney S. (2024). A long-acting LEAP2 analog reduces hepatic steatosis and inflammation and causes marked weight loss in mice. Mol. Metab..

[45] Dai J., Zhang L., Zhang R., Ge J., Yao F., Zhou S. (2024). Hepatocyte deubiquitinating enzyme OTUD5 deficiency is a key aggravator for metabolic dysfunction-associated steatohepatitis by disturbing mitochondrial homeostasis. Cell. Mol. Gastroenterol. Hepatol..

[46] Fondevila M.F., Novoa E., Fernandez U., Dorta V., Parracho T., Kreimeyer H. (2024). Inhibition of hepatic p63 ameliorates steatohepatitis with fibrosis in mice. Mol. Metab..

