# Supplementary material for: Metallothionein 1B attenuates inflammation and hepatic steatosis in MASH by inhibiting the AKT/PI3K pathway
Source: J Lipid Res. 2024 Nov 16;66(1):100701. doi: 10.1016/j.jlr.2024.100701 (PMC11714418; doi:10.1016/j.jlr.2024.100701)
Supplement: Supplemental figures [file mmc1.doc]

**
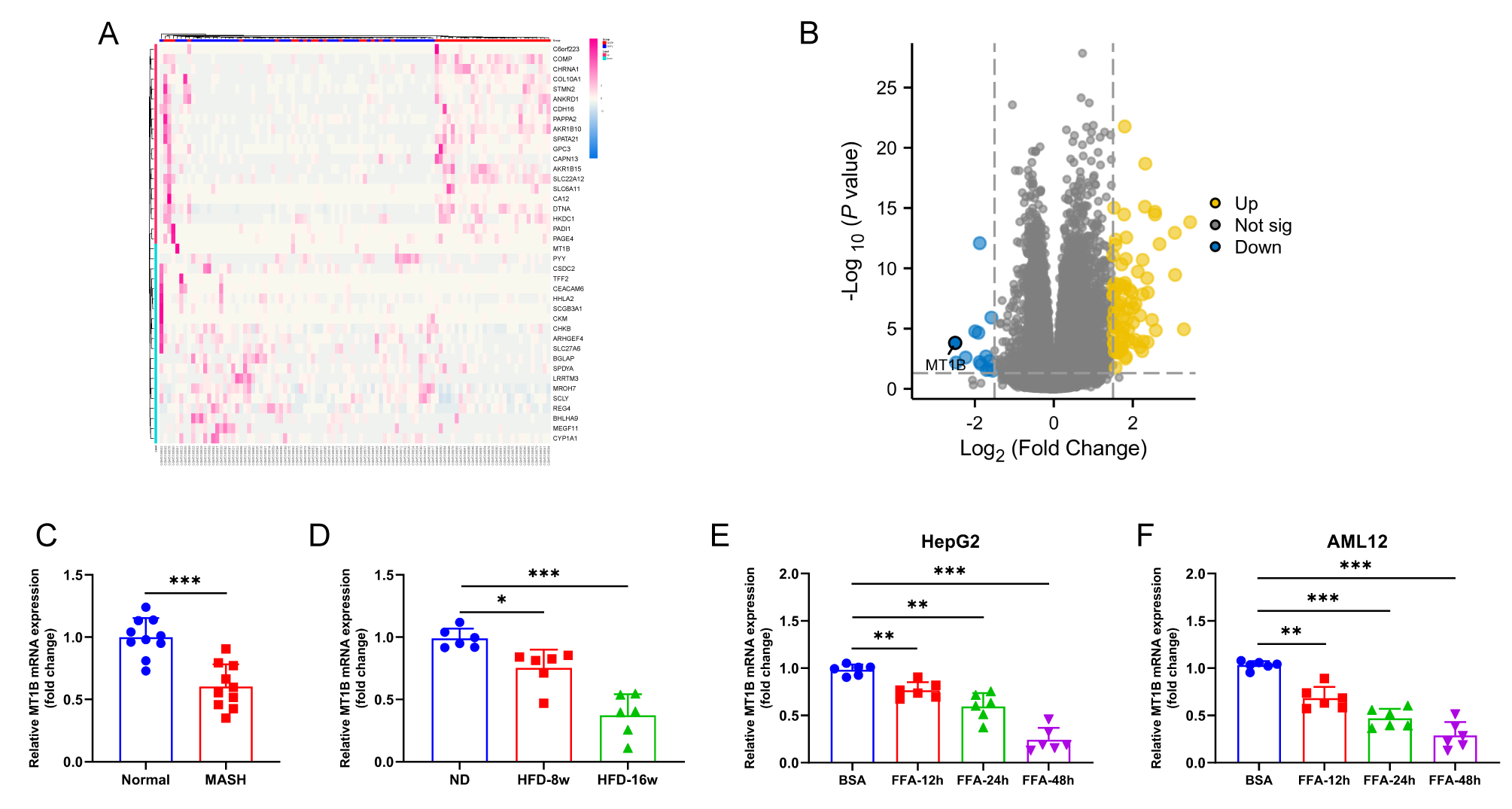
**

**Supplemental Fig. 1.** Bioinformatics prediction and qRT-PCR detection of MT1B expression. A: Volcano plot from the GSE167523 database shows significantly lower MT1B mRNA expression in MASH tissue samples compared to normal tissues. B: Heatmap illustrating the differential expression of MT1B between MASH and normal tissue samples. C: qRT-PCR results indicate that MT1B mRNA expression is significantly lower in liver tissues from MASH patients compared to normal liver tissues (n = 6). D: In HFD-induced mouse models, MT1B mRNA expression significantly decreased as MASH progressed (n = 6). E, F: MT1B mRNA expression levels in HepG2 and AML12 cells gradually decreased with increasing FFA induction time (n = 6). Data are represented as mean±SD. Data in (C) were presented by two-tailed Student's *t*-test. Data in (D-F) were presented by one-way ANOVA test. **P*<0.05, ***P*<0.01, ****P*<0.001.

**
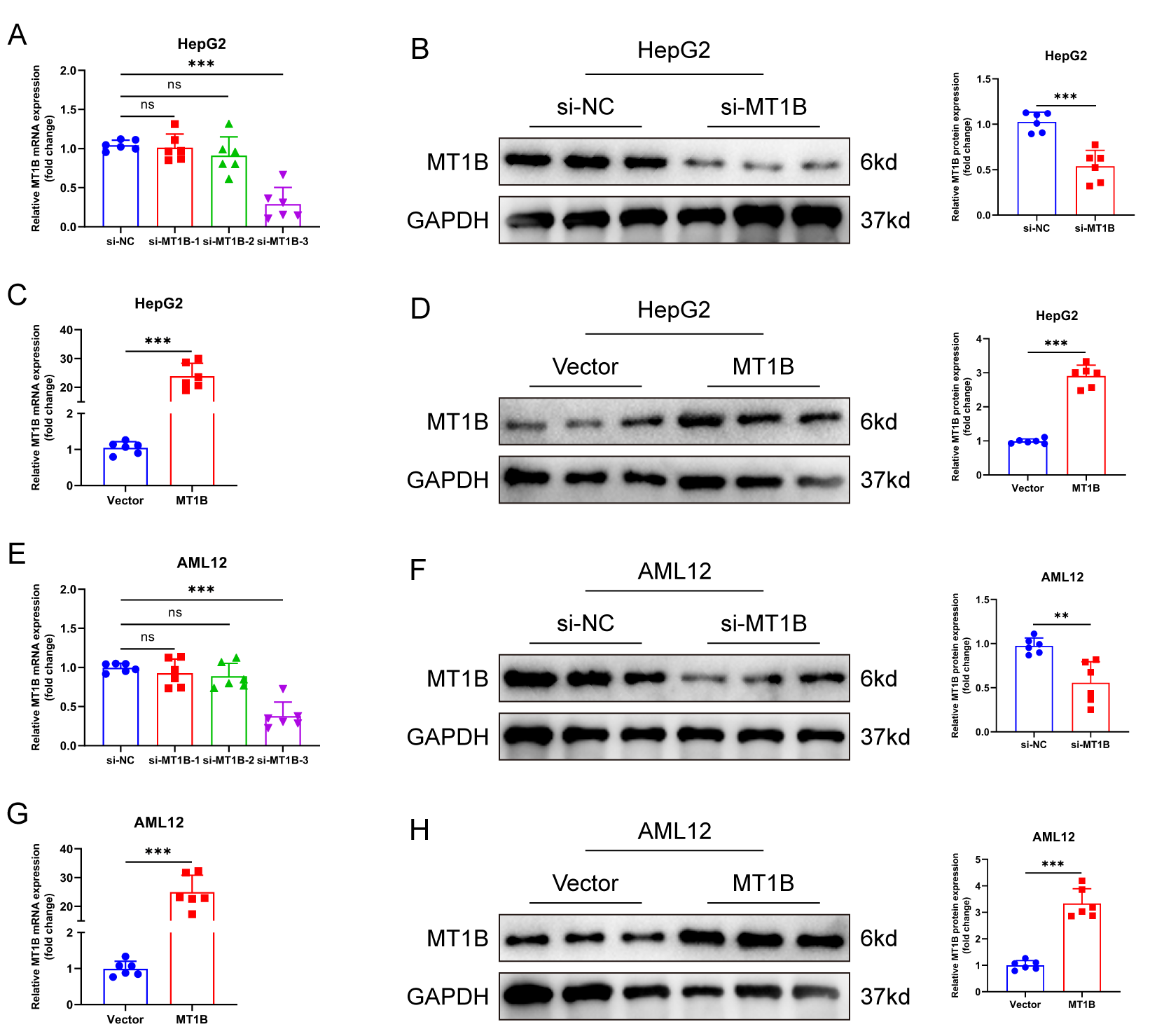
**

**Supplemental Fig. 2.** Efficiency verification of MT1B manipulation. A, B: qRT-PCR and western blot results confirm successful knockdown of MT1B in HepG2 cells (n = 6). C, D: qRT-PCR and western blot results confirm successful overexpression of MT1B in HepG2 cells (n = 6). E, F: qRT-PCR and western blot results confirm successful knockdown of MT1B in AML12 cells (n = 6). G, H: qRT-PCR and western blot results confirm successful overexpression of MT1B in AML12 cells (n = 6). Data are represented as mean±SD. Data in (A, E) were presented by one-way ANOVA test. Data in (B-D, F-H) were presented by two-tailed Student's *t*-test. ***P*<0.01, ****P*<0.001.

**
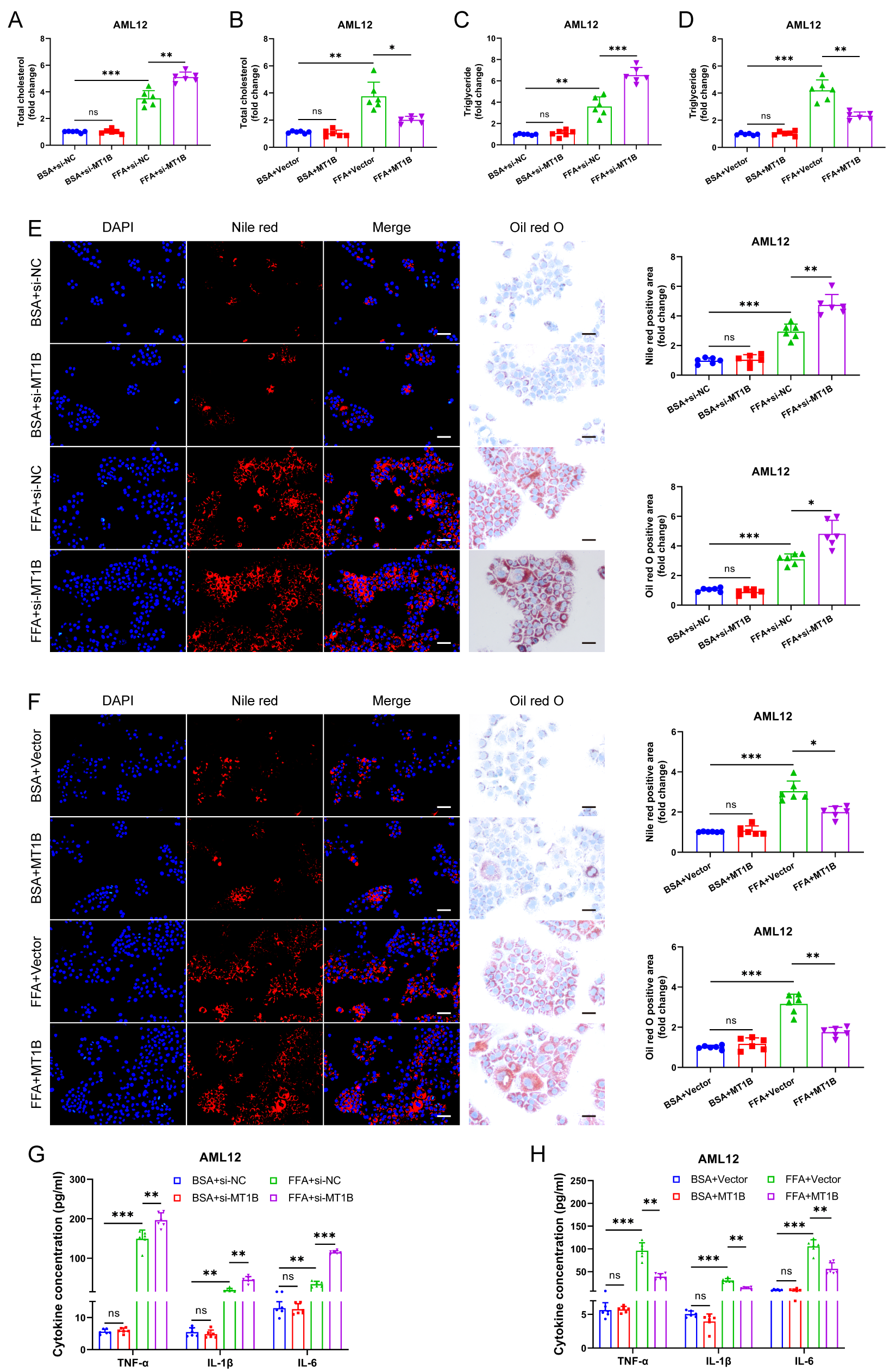
**

**Supplemental Fig. 3.** Regulation of lipid metabolism and inflammation by MT1B in AML12 cells. A, B: TG levels increased in MT1B-knockdown and decreased in MT1B-overexpressing AML12 cells (n = 6). C, D: TC levels increased in MT1B-knockdown and decreased in MT1B-overexpressing AML12 cells (n = 6). E, F: Nile red and Oil red O staining shows that the number and size of lipid droplets significantly increased in MT1B-knockdown cells and decreased in MT1B-overexpressing cells (n = 6). G: ELISA shows that the expression of pro-inflammatory factors (TNF-α, IL-6, IL-1β) was significantly upregulated in MT1B-knockdown AML12 cells (n = 6). H: The expression of pro-inflammatory factors was significantly downregulated and the anti-inflammatory factor increased in MT1B-overexpressing AML12 cells (n = 6). Data in (A-F) were presented by one-way ANOVA test. Data in (G, H) were presented by two-way ANOVA test. **P*<0.05, ***P*<0.01, ****P*<0.001.

**
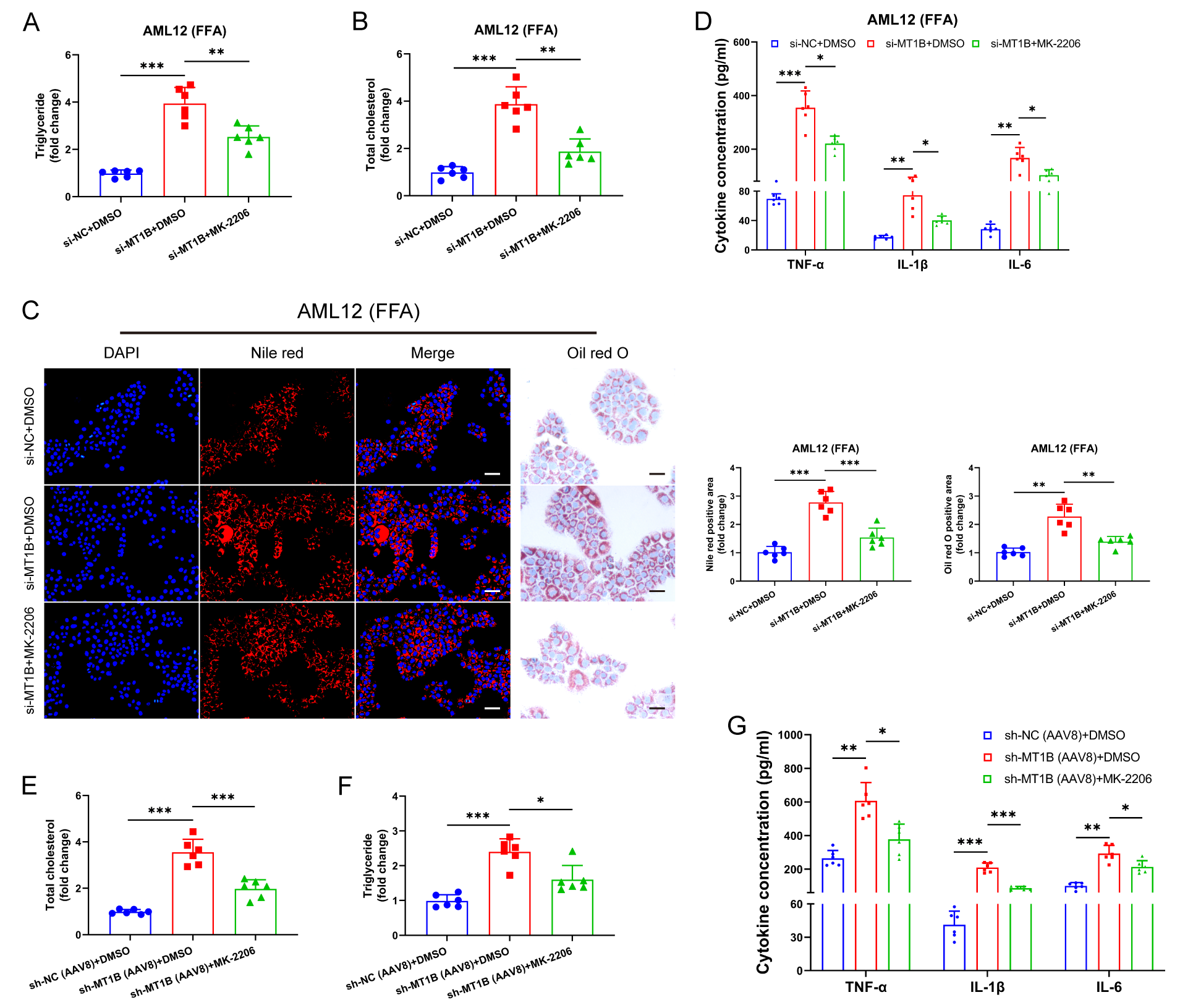
**

**Supplemental Fig. 4.** Further effects of AKT inhibitor on MT1B silencing in MASH. A, B: Quantitative analysis of TC and TG indicates that the AKT inhibitor (MK-2206) reversed the increase in TC and TG levels caused by MT1B silencing in AML12 cells (n = 6). C: Oil red O and Nile red staining show that MK-2206 reversed the increased lipid accumulation in MT1B-silenced AML12 cells (n = 6). D: The AKT inhibitor reversed the increase in pro-inflammatory factor expression caused by MT1B silencing in AML12 cells (n = 6). E, F: In MT1B-silenced mouse livers, the AKT inhibitor significantly reduced triglyceride and total cholesterol levels (n = 6). G: ELISA shows that the upregulation of pro-inflammatory factors (TNF-α, IL-6, IL-1β) in MT1B-silenced mouse livers was reversed by the AKT inhibitor (n = 6). Data in (A-C, E, F) were presented by one-way ANOVA test. Data in (D, G) were presented by two-way ANOVA test. **P*<0.05, ***P*<0.01, ****P*<0.001.

**
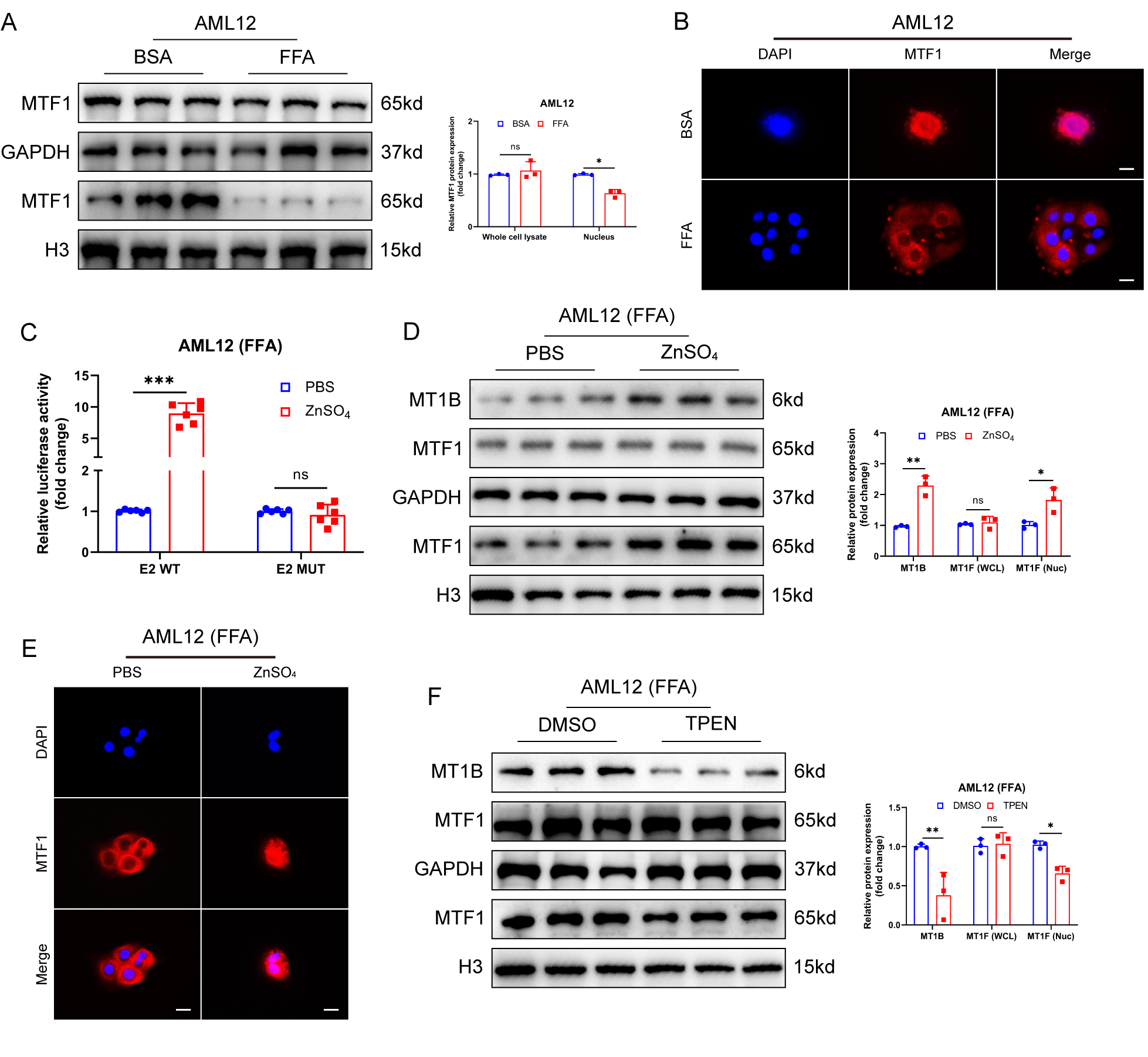
**

**Supplemental Fig. 5.** Further validation of Zn^2+^ upregulating MT1B through MTF1. A: Under FFA treatment, the total protein level of MTF1 did not change significantly, but the nuclear MTF1 level in AML12 cells was markedly reduced (n = 3). B: Immunofluorescence results show that FFA treatment significantly reduced the nuclear translocation of MTF1 in AML12 cells. C: Luciferase reporter assay indicates that Zn^2+^ treatment enhanced the binding activity of MTF1 to the MT1B promoter (n = 6). D, E: Western blot and immunofluorescence results demonstrate that Zn^2+^ treatment significantly increased the nuclear translocation of MTF1 in AML12 cells (n = 3).

F: Western blot results demonstrate that Zn^2+^ inhibitor (TPEN) significantly inhibited the nuclear translocation of MTF1 in AML12 cells (n = 3). Data are represented as mean±SD. Data in (A, C, D, F) were presented by two-way ANOVA test. **P*<0.05, ***P*<0.01, ****P*<0.001.


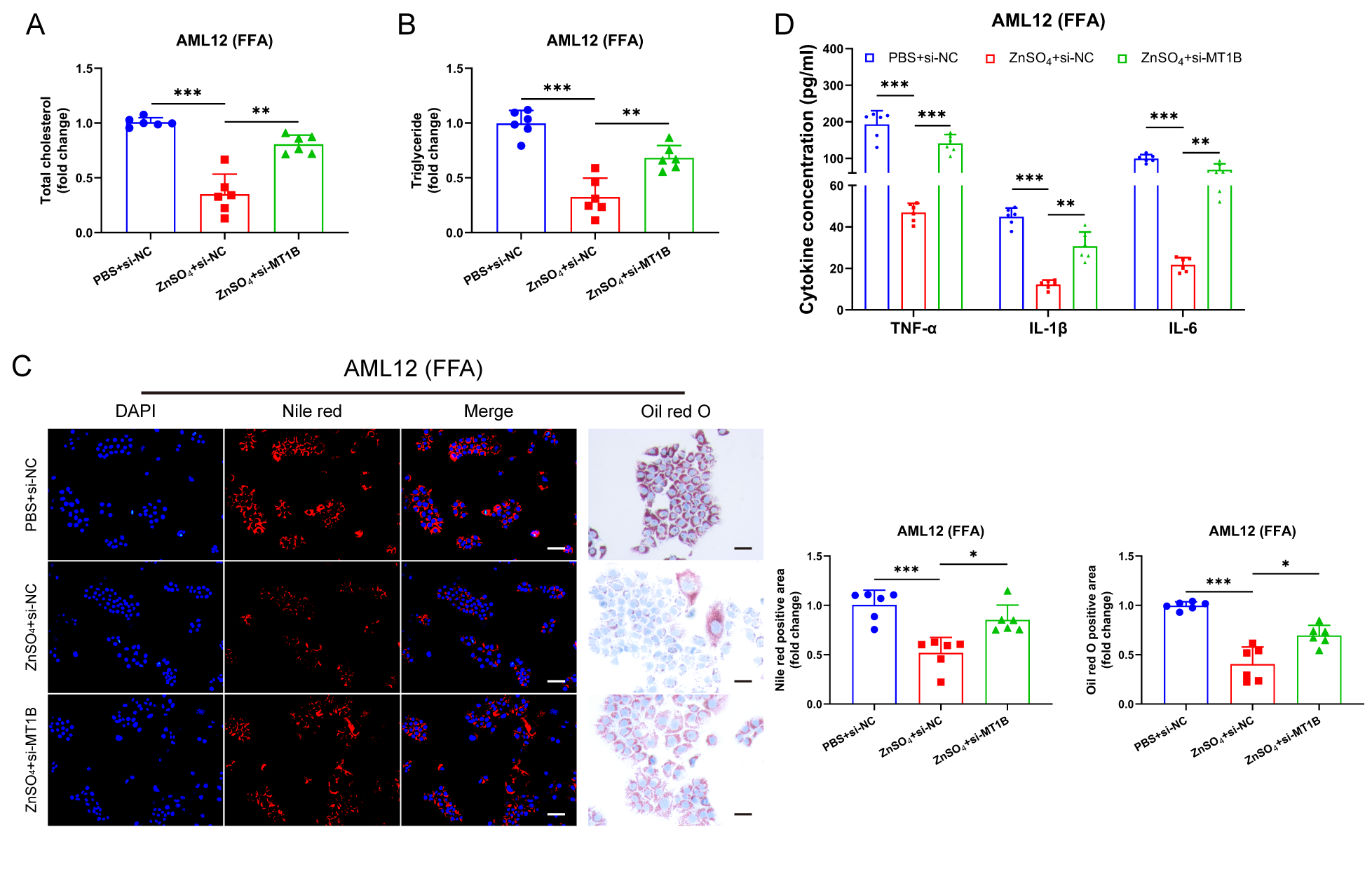


**Supplemental Fig. 6.** Zn^2+^ alleviate high-fat induced lipid metabolism disorder and inflammation in AML12 cells. A, B: Zn^2+^ treatment significantly alleviated TC and TG levels, but MT1B silencing reversed these effects (n = 6). C: Oil red O and Nile red staining show that MT1B-silenced reversed the decreased lipid accumulation in Zn^2+^ treatment AML12 cells (n = 6). D: ELISA results show that Zn^2+^ treatment significantly reduced the expression of pro-inflammatory factors (TNF-α, IL-6, IL-1β), but MT1B silencing reversed the anti-inflammatory effects of Zn^2+^ (n = 6). Data in (A-C) were presented by one-way ANOVA test. Data in (D) was presented by two-way ANOVA test. **P*<0.05, ***P*<0.01, ****P*<0.001.

**
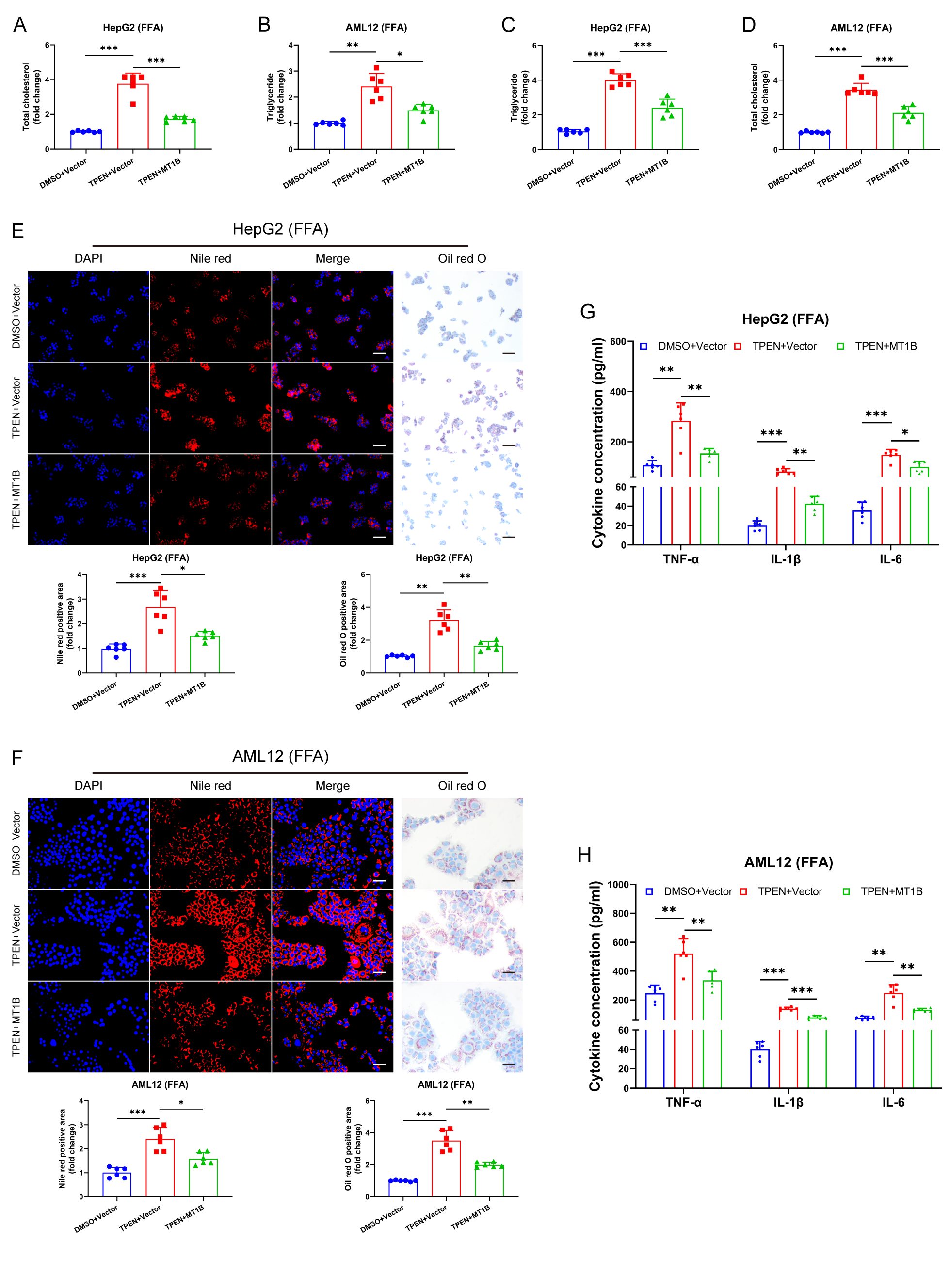
**

**Supplemental Fig. 7.** Zn^2+^ inhibitor (TPEN) can aggravate hyperlipid-induced lipid metabolism disorders and inflammation. A-D: Application of TPEN significantly increased TC and TG levels, but overexpression of MT1B reversed these effects (n = 6). E, F: Oil red O and Nile red staining show that upregulation of MT1B reversed increased lipid accumulation in TPEN-treated HepG2 and AML12 cells (n = 6). G, H: ELISA results show that TPEN treatment significantly up-regulated the expression of pro-inflammatory factors (TNF-α, IL-6, IL-1β), but MT1B overexpression could reverse the proinflammatory effect of TPEN (n = 6). Data in (A-F) were presented by one-way ANOVA test. Data in (G, H) were presented by two-way ANOVA test. **P*<0.05, ***P*<0.01, ****P*<0.001.
