# Supplementary material for: Metallothionein 1B attenuates inflammation and hepatic steatosis in MASH by inhibiting the AKT/PI3K pathway
Source: J Lipid Res. 2024 Nov 16;66(1):100701. doi: 10.1016/j.jlr.2024.100701 (PMC11714418; doi:10.1016/j.jlr.2024.100701)
Supplement: Supplemental tables [file mmc2.doc]

Supplementary Table 1 Clinicopathological characteristics of participants with normal and MASH patients.

| **Characteristics** | **Normal** | **MASH** | ***P* value** |
| --- | --- | --- | --- |
| Sex | Male | Male |  |
| Age (years) | 53.20 ± 4.61 | 51.20 ± 9.81 | 0.5668 |
| BMI (kg/m^2^) | 21.76 ± 2.98 | 27.93 ± 4.33 | 0.0016 |
| ALT (U/L) | 13.90 ± 2.98 | 30.10 ± 12.24 | 0.0014 |
| AST (U/L) | 18.70 ± 6.82 | 38.00 ± 16.92 | 0.0036 |
| TG (mmol/L) | 1.05 ± 0.31 | 1.54 ± 0.40 | 0.0065 |
| TC (mmol/L) | 4.15 ± 0.95 | 4.88 ± 0.88 | 0.0901 |
| Glucose (mmol/L) | 5.04 ± 0.87 | 6.33 ± 1.70 | 0.0466 |
| MT1B expression (mRNA) | 1.00 ± 0.15 | 0.63 ± 0.17 | 0.0001 |
| MT1B expression (protein) | 1.00 ± 0.28 | 0.56 ± 0.27 | 0.0025 |

**Supplemental Table 2 Primer sequences for qRT-PCR and siRNAs sequences.**

| Nucleic acids | Sequences |
| --- | --- |
| GAPDH | F: 5’-GAAGGTGAAGGTCGGAGT-3’ |
|  | R: 5’-GAAGATGGTGATGGGATTTC-3’ |
| MT1B | F: 5’-ACACAGTGTCCCTGGGTTAG-3’ |
|  | R: 5’-GACAGCCTCGATCCTCAGAA-3’ |
| E2 | F: 5’-AGGTGGGAAATCCAAAGGTCA-3’ |
|  | R: 5’-GAGCACAGGGAGAGAGTTGT-3’ |
| si-NC | 5’-UUCUCCGAACGUGUCACGU-3’ |
|  | 5’-ACGUGACACGUUCGGAGAA-3’ |
| si-MT1B-1 | 5’-GCAAGUGCAAAGAGUGCAAAU-3’ |
|  | 5’-UUGCACUCUUUGCACUUGCAG-3’ |
| si-MT1B-2 | 5’-AAAAUCCAGGUUAGUACUGGU-3’ |
|  | 5’-CAGUACUAACCUGGAUUUUUU-3’ |
| si-MT1B-3 | 5’-GCUCCUGCACCACAGGUAAGG-3’ |
|  | 5’-UUACCUGUGGUGCAGGAGCAG-3’ |

**Supplementary Table 3 Antibodies used for western bolt.**

| **Antibodies** | **Vendor** | **Catalog** | **Dilution ratio** |
| --- | --- | --- | --- |
| MT1B | LS Bio | LS-C497343 | WB: 1:2000 |
| GAPDH | Abcam | ab8245 | WB: 1:5000 |
| Histone H3 | Abcam | ab1791 | WB: 1:4000 |
| AKT | Proteintech | 10176-2-AP | WB: 1:6000 |
| p-AKT (T308) | Proteintech | 29163-1-AP | WB: 1:1000 |
| p-AKT (S473) | Proteintech | 66444-1-Ig | WB: 1:2000 |
| PI3K (p85) | CST | 4292 | WB: 1:1000 |
| p-PI3K | CST | 4228 | WB: 1:1000 |
| PDK1 | Proteintech | 18262-1-AP | WB: 1:2000 |
| p-PDK1 | CST | 3438 | WB: 1:1000 |
| MTF1 | Proteintech | 25383-1-AP | WB: 1:2000  IF: 1:100 |
| HRP-conjugated Goat Anti-Mouse IgG | Proteintech | SA00001-1 | WB: 1:5000 |
| HRP-conjugated Goat Anti-Rabbit IgG | Proteintech | SA00001-2 | WB: 1:5000 |
| CoraLite594-conjugated Goat Anti-Rabbit IgG | Proteintech | SA00013-4 | IF: 1:500 |
